# A hybrid Lagrangian–Eulerian model for vector-borne diseases

**DOI:** 10.1007/s00285-024-02109-5

**Published:** 2024-06-18

**Authors:** Daozhou Gao, Xiaoyan Yuan

**Affiliations:** 1 Department of Mathematics and Statistics, Cleveland State University, Cleveland, OH 44115 USA; 2 Department of Mathematics, Shanghai Normal University, Shanghai, 200234 China

**Keywords:** Vector–borne disease, Basic reproduction number, Population movement, Lagrangian approach, Eulerian approach, Optimal vector control, 92D30, 34D23, 91D25, 15A42

## Abstract

In this paper, a multi-patch and multi-group vector-borne disease model is proposed to study the effects of host commuting (Lagrangian approach) and/or vector migration (Eulerian approach) on disease spread. We first define the basic reproduction number of the model, $\mathcal {R}_0$, which completely determines the global dynamics of the model system. Namely, if $\mathcal {R}_0 \le 1$, then the disease–free equilibrium is globally asymptotically stable, and if $\mathcal {R}_0 > 1$, then there exists a unique endemic equilibrium which is globally asymptotically stable. Then, we show that the basic reproduction number has lower and upper bounds which are independent of the host residence times matrix and the vector migration matrix. In particular, nonhomogeneous mixing of hosts and vectors in a homogeneous environment generally increases disease persistence and the basic reproduction number of the model attains its minimum when the distributions of hosts and vectors are proportional. Moreover, $\mathcal {R}_0$ can also be estimated by the basic reproduction numbers of disconnected patches if the environment is homogeneous. The optimal vector control strategy is obtained for a special scenario. In the two-patch and two-group case, we numerically analyze the dependence of the basic reproduction number and the total number of infected people on the host residence times matrix and illustrate the optimal vector control strategy in homogeneous and heterogeneous environments.

## Introduction

Vector-borne diseases are diseases primarily transmitted to humans and other animals by blood-feeding arthropods such as mosquitoes, ticks, and bugs and caused by pathogens such as bacteria, viruses, and parasites. The diseases are prevalent in tropical and subtropical regions, accounting for more than 17$\%$ of all infectious diseases. Common vector-borne diseases, including malaria, dengue fever, schistosomiasis, Chagas disease, leishmaniasis, Japanese encephalitis, and onchocerciasis, result in over 700,000 deaths annually (World Health Organization 2020b). For example, the World Health Organization estimated that there were 247 million malaria cases and 619,000 deaths globally in 2021 (World Health Organization 2022). The number of dengue cases has been increasing rapidly over the past two decades, from 0.5 million in 2000 to 5.2 million in 2019 (World Health Organization 2023b). The Americas region alone reported 2.7 million dengue cases and 1206 deaths from January to October 2019, of which over 22,000 were classified as severe dengue (World Health Organization 2019b). There are an estimated 6–7 million cases of Chagas disease worldwide, mostly in Latin America (World Health Organization 2023a). Japanese encephalitis is the main cause of viral encephalitis in many Asian countries, with an estimated 68,000 clinical cases every year (World Health Organization 2019a).

In recent decades, factors such as globalization, urbanization, and transportation modernization have greatly enhanced regional and global connectivity. The annual number of inbound and outbound tourists in China has exceeded 100 million, and the country has an estimated floating population of 493 million (National Bureau of Statistics of China 2021). The frequent population movement leads to the rapid spread of infectious diseases, which brings great challenges to disease prevention and control. After the first discovery of Chikungunya virus in Tanzania in 1952, the virus caused sporadic outbreaks in sub-Saharan Africa and Southeast Asia. However, since 2004, Chikungunya fever has swiftly spread to more than 60 countries in Africa, Asia, America and Europe (World Health Organization 2020a). The first autochthonous cases of Zika fever in Brazil were confirmed in May 2015. Subsequently, evidence of mosquito-acquired Zika virus infections has appeared in new regions, including South and Central America and the Caribbean. So far, a total of 86 countries and regions have reported cases of Zika virus disease (World Health Organization 2021). Therefore, it is crucial to consider the role of movement on disease propagation, which is helpful to understand the mechanism of spatial transmission, assess the risk of cross-regional (such as inter-country, inter-city) disease spread, and design effective prevention and control measures.

Mathematical modeling of vector–borne diseases has a long history. The well-known Ross–Macdonald model for malaria was initially proposed by Ross (1911) in 1911, and later extended by Macdonald in the 1950s (Macdonald 1957). Over the past few decades, various biological, epidemiological, immunological, and socioeconomic factors have been incorporated into the model for malaria and other mosquito-borne or vector-borne diseases (Feng and Velasco-Hernández 1997; Gao et al. 2016; Lou and Wu 2017; Reiner et al. 2013; Wu et al. 2020). Among them, an increasing number of patch models for vector-borne diseases have been developed to describe disease spread in discrete space (Arino 2009). Inspired by fluid mechanics, Cosner et al. (2009) classified epidemic patch models into two types based on description of movement. One is the Lagrangian type which imitates human commuting behavior. Individuals are identified as resident of a given patch or group and they may visit other patches where they can get infected or infect others but their identity remains the same. The other is the Eulerian type that imitates human migration. Individuals belong to the patch where they are located and they can migrate to other patches and become members of the immigrated patches.

Since Lagrangian models are essentially a class of multi-group models, the development and application of Lagrangian epidemic models can be traced back to the work of Rushton and Mautner (1955), Lajmanovich and Yorke (1976), Post et al. (1983), and Sattenspiel and Dietz (1995). To the best of the authors’ knowledge, based on the Ross–Macdonald model, Dye and Hasibeder (1986), Hasibeder and Dye (1988) established the first Lagrangian vector-borne disease model in which only vectors commute in a homogeneous patchy environment. Torres-Sorando and Rodríguez (1997) considered the two types of host mobility patterns and compared them in terms of the time elapsed until reaching equilibrium and equilibrium prevalence. Cosner et al. (2009) constructed both Lagrangian and Eulerian models to examine the effects of human and mosquito movements on vector-borne disease dynamics in heterogeneous environments. Lee and Castillo-Chavez (2015) developed a two-patch dengue model (SEIR structure for humans and SEI structure for mosquitoes) with bilinear incidence and applied optimal control theory to minimize dengue prevalence in hosts and vectors at a minimal cost. Bichara et al. (2016) considered a similar dengue model but with standard incidence and vertical transmission in vectors. Iggidr et al. (2016) generalized the Bailey–Dietz model (SIR-SI structure) to a multi-group model. They characterized the irreducibility of the host-vector contact network and showed the global dynamics of the model system. Ruktanonchai et al. (2016) considered a modified Lagrangian Ross–Macdonald malaria model with mobile hosts and explored how to identify patches that are transmission foci. Bichara and Castillo-Chavez (2016) proposed a multi-patch and multi-group modeling framework that takes host effective population size into consideration and decouples host group from vector patch. After these, Moreno et al. (2017) and Zhang et al. (2018) used Lagrangian approach in modeling the geographical spread of Zika virus and West Nile virus, respectively. Recently, Soriano-Paños et al. (2020) elaborated a metapopulation model for the transmission of vector-borne diseases using a Markovian formalism where humans commute between patches daily.

There are quite a few vector-borne disease models using the Eulerian approach. Auger et al. (2008) proposed a multi-patch Ross–Macdonald model in which only hosts migrate between patches, and showed its threshold behavior. Some factors like competition between strains (Qiu et al. 2013), seasonality (Gao et al. 2014), almost periodicity and stage structure (Wang et al. 2020), heterogeneity in travel frequency (Chen and Gao 2020), and host vital dynamics (Saucedo and Tien 2022), are directly added to the multi-patch Ross–Macdonald model. Gao and Ruan (2012) modeled malaria spread between patches with human and mosquito migration, intrinsic and extrinsic incubation periods, acquired immunity of humans, and logistic growth of humans and mosquitoes and studied the impact of population dispersal on disease persistence. Arino et al. (2012) included partial immunity of humans, general biting rate, and constant recruitment of humans and mosquitoes into their multi-patch malaria model and identified the reservoirs of infection. Xiao and Zou (2014) derived a delay differential equations patch model with fixed latencies in both hosts and vectors. Mukhtar et al. (2020) formulated a metapopulation malaria model by adding asymptomatic infection and transmission and fitted the model to the weekly case data in South Sudan. Multi-patch models with host migration are also developed to study the spread of Rift Valley fever (Gao et al. 2013; Xue et al. 2012), West Nile fever (Liu et al. 2006), Zika virus disease (Harvim et al. 2019), dengue fever (Mishra and Gakkhar 2018), and tick-borne disease (Gaff and Gross 2007; Zhang et al. 2021). Interestingly, Iggidr et al. (2017) derived a Bailey-Dietz type model using an idea similar to that of Sattenspiel and Dietz (1995), from which they arrived at a Lagrangian multi-group model. The interested reader may refer to the introduction of the paper by Gao and Cao (2024) for multi-patch models on directly transmitted diseases.

Most spatial vector-borne disease models only consider host movement (Arino et al. 2012; Auger et al. 2008; Bichara and Castillo-Chavez 2016; Gao et al. 2014; Mukhtar et al. 2020; Qiu et al. 2013; Saucedo and Tien 2022; Torres-Sorando and Rodríguez 1997; Xiao and Zou 2014; Zhang et al. 2018), while a few involve movements of both hosts and vectors using Lagrangian approach (Cosner et al. 2009; Gao and Cao 2024; Iggidr et al. 2016) or Eulerian approach (Cosner et al. 2009; Gao and Ruan 2012). From the descriptions of Lagrangian and Eulerian approaches, one can see that the former is suitable for small geographical scales, while the latter works on large scales. Mark-release-recapture experiments reveal that mosquitoes only have limited mobility (Muir and Kay 1998). The maximum flight distance is between 50 m and 50 km, and the average flight distance is between 25 m and 6 km, varying among different mosquito species (Verdonschot and Besse-Lototskaya 2014). For example, the primary vectors of malaria parasites, *Anopheles* mosquitoes, can fly an average maximum distance of 3.49 km. However, the yellow fever mosquitoes, *Aedes aegypti*, only travel between 100–200 m (Russell et al. 2005). There is little evidence to support the idea of a memorized home range between feeding and oviposition sites. Mosquitoes are not typically considered territorial. Their travel behavior is more closely tied to their need for blood sources and breeding sites. They may disperse short distances to avoid overcrowded habitats. Therefore, it is appropriate to describe vector movement by the Eulerian approach and host movement by the Lagrangian approach when a small and medium-sized patchy environment is concerned.

In the next section, we formulate a mixed vector-borne disease model in which the movements of vectors and hosts follow Eulerian and Lagrangian approaches, respectively. In Sect. 3, we compute the basic reproduction number of the model and establish the global dynamics of the model system. In Sect. 4, lower and upper bounds on the basic reproduction number that are independent or dependent of host and vector movements are obtained. Moreover, we consider how to allocate limited resources for vector control to minimize the reproduction number. In Sect. 5, we numerically investigate the effects of varying residence times on disease persistence and host prevalence. Finally, we summarize the main findings of the current study and discuss some future research directions.

## Model formulation

We aim to develop a multi-patch and multi-group vector-host disease model where hosts commute and vectors migrate between patches. The total hosts are divided into *m* groups in terms of age, gender, occupation, residence, etc, while the total vectors are divided into *n* patches in terms of its present location. Like Bichara and Castillo-Chavez (2016), and Gao and Cao (2024), the structure of the host groups is decoupled to that of vector patches. Using the single patch Ross-Macdonald model as a building block, we make the following assumptions:

1. Host births and deaths are not taken into account.
2. Vector birth and death rates in each patch are balanced but vary by patch.
3. Mosquito biting rate depends on patch.
4. Both transmission probabilities from an infected vector to a susceptible host and from an infected host to a susceptible vector depend on which group the host belongs to.
5. Host movement is Lagrangian and vector movement is Eulerian.
6. Hosts spend their full time in the specified patchy environment and there is no birth or death for vectors during travel.
7. Disease states of hosts and vector do not affect their travel behavior.

The set of host groups and vector patches are denoted by $\Omega _h=\{1,2,...,m\}$ and $\Omega _v=\{1,2,...,n\}$, respectively. The total population of host group $i\in \Omega _h$ at time *t*, denoted by $H_i(t)$, is split into susceptible hosts $S_i^h(t)$ and infected hosts $I_i^h(t)$. Similarly, the total population of vectors in patch $j\in \Omega _v$ at time *t*, denoted by $V_j(t)$, is split into susceptible vectors $S_j^v(t)$ and infected vectors $I_j^v(t)$. Thus, we have

$$\begin{aligned} H_i(t)=S_i^h(t)+I_i^h(t)\ \text{ and } \ V_j(t)=S_j^v(t)+I_j^v(t),\quad i\in \Omega _h,\ j\in \Omega _v. \end{aligned}$$

Let $a_j$ denote the number of bites per vector per unit time in patch *j*, $b_i$ the transmission probability from an infectious vector to a susceptible host of group *i* per bite, $c_i$ the transmission probability from an infectious host of group *i* to a susceptible vector per bite, $\gamma _i$ the host recovery rate of group *i*, and $\mu _j$ the birth and death rates of vectors in patch *j*. The parameters $a_j$, $b_i$, $c_i$, $\gamma _i$ and $\mu _j$ are positive for all $i \in \Omega _h$ and $j \in \Omega _v$.

Denote the residence times matrix of hosts by $P=(p_{ik})_{m \times n}$, where $p_{ik}$ is the proportion of time that hosts of group $i\in \Omega _h$ stay in patch $k\in \Omega _v$. The travel rates matrix of vectors is labeled by $D=(d_{jr})_{n \times n}$, where $d_{jr}$ is the migration rate of vectors from patch *r* to patch *j*. By assumption (6), the two matrices satisfy

$$\begin{aligned} 0 \le p_{ik} \le 1\quad \textrm{and}\quad \sum _{k\in \Omega _{v}}p_{ik}=1, \quad i\in \Omega _h,\, k \in \Omega _v, \end{aligned}$$

and

$$\begin{aligned} d_{jr} \ge 0,\quad j \ne r \quad \textrm{and}\quad -d_{rr}=\sum _{ j \ne r} d_{jr}, \quad j,\, r \in \Omega _v. \end{aligned}$$

For the convenience of mathematical analysis, we make an additional assumption:

($$\mathcal {H}1$$)
The travel rates matrix of vectors $D=(d_{jr})_{n \times n}$ is irreducible.

A square matrix is irreducible if it is not similar to an upper triangular matrix via a permutation. Moreover, it is irreducible if and only if its associated directed graph is strongly connected (Horn and Johnson 2013). The irreducibility of the matrix *D* means that if there are vectors in one patch then there are vectors in all patches, which ensures that the disease can spread across all patches. We denote the set of patches with host visits by

$$\begin{aligned} \Omega _v^0=\{j\in \Omega _v \; | \; p_{ij}>0\ \text{ for } \text{ some } \ i \in \Omega _h\}. \end{aligned}$$

Clearly, $\Omega _{v}^{0}\subseteq \Omega _{v}$. Since patch $j\in \Omega _{v}\backslash \Omega _{v}^{0}$ is host-free, no new infections appear in the patch. However, patch *j* is not disease-free due to the migration of infected vectors from other patches.

**Fig. 1:**
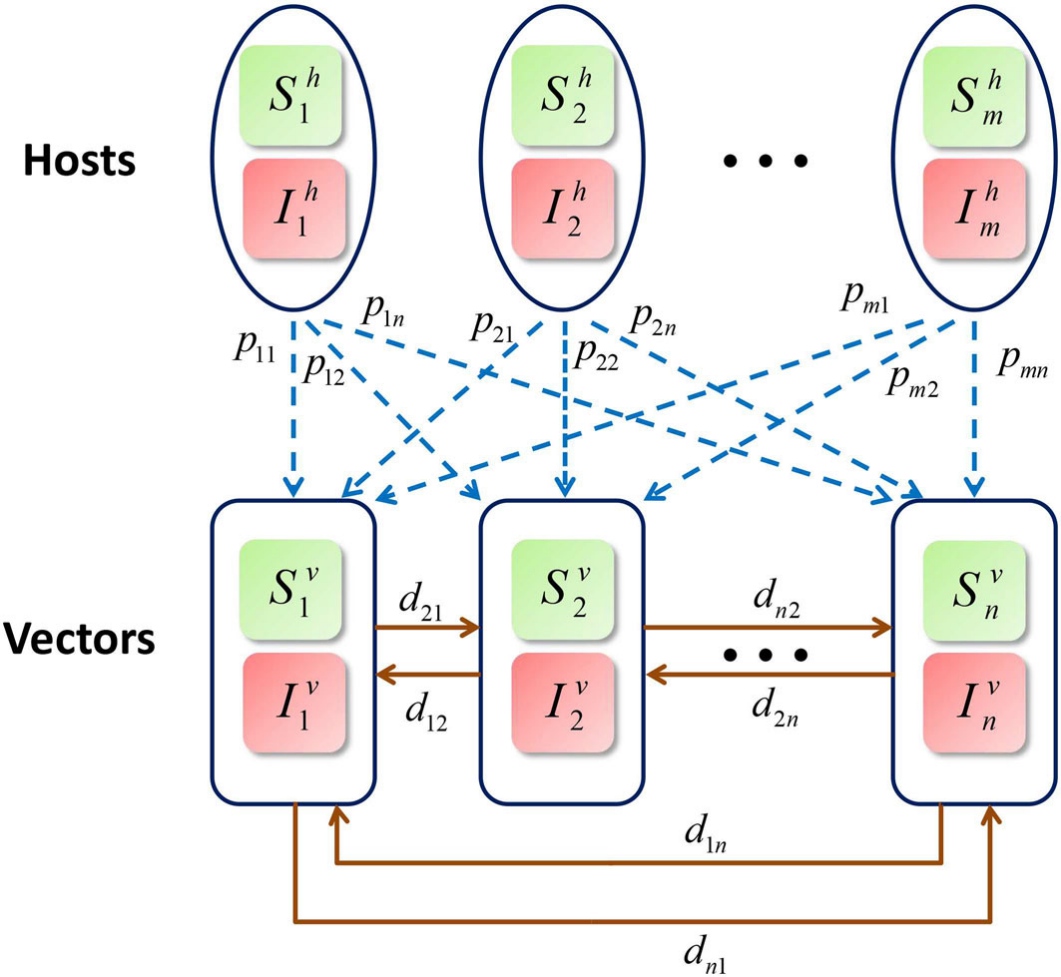
Flow chart of the Lagrangian–Eulerian vector-borne disease model

Next, we adopt the concept of effective (infected) population size (Bichara and Castillo-Chavez 2016; Bichara et al. 2015) to derive the forces of infection of hosts and vectors. In patch $k\in \Omega _{v}^{0}$, the total number of vectors is $V_k$ of which there are $I_k^v$ infected vectors. Meanwhile, the average number of hosts of group *l* who are currently in patch *k* is $p_{lk}H_l$, so the total host population size in patch *k* is $\sum _{l \in \Omega _h}p_{lk}H_l$. Then the number of bites per host received per unit time in patch *k* is

$$\begin{aligned} \frac{a_{k}V_k}{\sum _{l \in \Omega _h}p_{lk}H_l}. \end{aligned}$$

Thus, the force of infection for host group *i* in patch $k\in \Omega _{v}^{0}$ is given by

$$\begin{aligned} b_i \frac{a_k V_k}{\sum _{l \in \Omega _h}p_{lk}H_l} \frac{I_k^v}{V_k} = b_i a_{k} \frac{I_k^v}{\sum _{l \in \Omega _h}p_{lk}H_l}, \end{aligned}$$

and the force of infection for vectors in patch $j\in \Omega _v^0$ is expressed as

$$\begin{aligned} \sum _{i \in \Omega _h} c_i a_j \frac{ p_{ij}I_i^h}{\sum _{l \in \Omega _h}p_{lj}H_l} =a_j \frac{\sum _{i \in \Omega _h}c_ip_{ij}I_i^h}{\sum _{l \in \Omega _h}p_{lj}H_l}. \end{aligned}$$

Following the above assumptions and the flow chart in Fig. 1, the vector-host interaction can be described by the following $2(m+n)$-dimensional system

2.1 $$\begin{aligned} \begin{aligned} \frac{dS_i^h}{dt}&=-b_i \sum _{k \in \Omega _v^0} a_{k} \frac{I_k^v}{\sum _{l \in \Omega _h}p_{lk}H_l} p_{ik} S_i^h + \gamma _i I_i^h,\quad i \in \Omega _h, \\ \frac{dI_i^h}{dt}&=b_i \sum _{k \in \Omega _v^0} a_{k} \frac{I_k^v}{\sum _{l \in \Omega _h}p_{lk}H_l} p_{ik} S_i^h - \gamma _i I_i^h,\quad i \in \Omega _h, \\ \frac{dS_j^v}{dt}&=\mu _j V_j -a_j \frac{\sum _{i \in \Omega _h}c_ip_{ij}I_i^h}{\sum _{l \in \Omega _h}p_{lj}H_l} S_j^v - \mu _j S_j^v + \sum _{r \in \Omega _v} d_{jr} S_r^v,\quad j \in \Omega _v, \\ \frac{dI_j^v}{dt}&=a_j \frac{\sum _{i \in \Omega _h}c_ip_{ij}I_i^h}{\sum _{l \in \Omega _h}p_{lj}H_l} S_j^v - \mu _j I_j^v + \sum _{r \in \Omega _v} d_{jr} I_r^v,\quad j \in \Omega _v, \end{aligned} \end{aligned}$$

with nonnegative initial conditions satisfying

$$\begin{aligned} H_i(0)=S_i^h(0)+I_i^h(0)>0,\ \ \forall \ i\in \Omega _h\ \ \text{ and } \ \ \sum \nolimits _{j\in \Omega _v} V_j(0)=V>0. \end{aligned}$$

Note that if $j\in \Omega _v\backslash \Omega _v^0$, i.e., no host on patch *j*, then the denominator and numerator of the force of infection for vectors in patch *j* equal zero. In this case, it is natural to set the corresponding infection term to zero.

The sum of the first two equations in (2.1) indicates that $H_i(t)\equiv H_i(0)$ for all $i\in \Omega _h$, i.e., the total population size of each host group is fixed. Adding the last two equations of (2.1) gives

2.2 $$\begin{aligned} \begin{aligned} \frac{dV_j}{dt}= \sum _{r \in \Omega _v} d_{jr} V_r, \quad j \in \Omega _v. \end{aligned} \end{aligned}$$

The irreducibility and essential nonnegativity of the travel rates matrix $D=(d_{jr})_{n \times n}$ implies that the vector migration model (2.2) has a unique positive equilibrium $\varvec{V}^*=(V_1^*,\dots ,V_n^*)$, which is globally asymptotically stable (see e.g., Gao et al. (2019)). Here $\varvec{V}^*$ is the unique positive solution to

$$\begin{aligned} \sum _{r \in \Omega _v} d_{jr} V_r=0,\ j \in \Omega _v \quad \textrm{and} \quad \sum _{r \in \Omega _v}V_r =\sum _{r \in \Omega _v}V_r(0)=V > 0. \end{aligned}$$

In other words, $(V_1^*,\dots ,V_n^*)^{\textrm{T}}$ is the normalized positive right eigenvector of *D* corresponding to the eigenvalue zero. Applying the theory of asymptotically autonomous systems (Castillo-Chavez and Thieme 1995; Zhao 2017), it follows from $V_j(t)\rightarrow V_j^*$ as $t\rightarrow \infty$ for all $j\in \Omega _v$ that the model system (2.1) is topologically equivalent to

2.3 $$\begin{aligned} \begin{aligned} \frac{dI_i^h}{dt}&=b_i \sum _{k \in \Omega _v^0} a_k \frac{I_k^v}{\sum _{l \in \Omega _h}p_{lk}H_l} p_{ik} \left( H_i - I_i^h \right) - \gamma _i I_i^h,\quad i \in \Omega _h, \\ \frac{dI_j^v}{dt}&=a_j \frac{\sum _{i \in \Omega _h}c_ip_{ij}I_i^h}{\sum _{l \in \Omega _h}p_{lj}H_l} \left( V_j^* - I_j^v \right) - \mu _j I_j^v + \sum _{r \in \Omega _v} d_{jr} I_r^v,\quad j \in \Omega _v. \\ \end{aligned} \end{aligned}$$

The model (2.3) is mathematically well-posed and biologically meaningful.

### Proposition 2.1

For any initial condition lying in

$$\begin{aligned} \begin{aligned} \Gamma =\{(I_1^h,\,\dots ,\,I_m^h,\,I_1^v,\,\dots ,\,I_n^v)\in {\mathbb {R}}^{m+n}_+ \mid I_i^h\le H_i,\,I_j^v\le V_j^*,\, i\in \Omega _{h},\,j\in \Omega _{v}\}, \end{aligned} \end{aligned}$$

the model (2.3) has a unique nonnegative solution for all time $t\ge 0$. Moreover, the domain $\Gamma$ is positively invariant with respect to (2.3).

### Proof

The continuous differentiability of the vector field generated by the right hand side of system (2.3) on $\Gamma$ implies that the system has a unique local solution. If $I_i^h=0$, then $dI_{i}^{h}/dt \ge 0$. Similarly, if $I_j^v=0$, then $dI_j^v/dt \ge 0$. If $I_i^h=H_i$ for some $i \in \Omega _h$, then $dI_i^h/dt =-\gamma _i H_i<0$. Similarly, if $I_j^v=V_j^*$ for some $j\in \Omega _v$, then

$$\begin{aligned} \begin{aligned} \frac{dI_j^v}{dt}&=-\mu _j V_j^*+\sum _{r\ne j} d_{jr} I_r^v+d_{jj} V_j^*=-\mu _j V_j^*+\sum _{r\ne j} d_{jr} I_r^v-\sum _{r\ne j} d_{jr} V_r^* \\&=-\mu _j V_j^*-\sum _{r\ne j} d_{jr} (V_r^*-I_r^v) \le -\mu _j V_j^*<0. \end{aligned} \end{aligned}$$

Therefore, $\Gamma$ is a positively invariant set of system (2.3). Since the invariant set $\Gamma$ is bounded, any solution starting in $\Gamma$ is always extendable and so must exist globally. $\square$

## Threshold dynamics

In this section, we first calculate the basic reproduction number of model (2.3), then obtain the global dynamic behavior of the model, and finally apply the result to the disconnected patch case.

### Basic reproduction number

The model (2.3) has a unique disease-free equilibrium $E_0=(0,\dots ,0,0,\dots ,0)$. Following the method of the next generation matrix (Diekmann et al. 1990; van den Driessche and Watmough 2002), the rates of appearance of new infections and transfer of individuals between compartments are

$$\begin{aligned}{} & {} \begin{aligned} \mathscr {F}&=\begin{pmatrix} b_1 \sum \limits _{k \in \Omega _v^0} a_k \dfrac{I_k^v}{\sum _{l \in \Omega _h} p_{lk} H_l} p_{1k} \left( H_1 - I_1^h \right) \\ \vdots \\ b_m \sum \limits _{k \in \Omega _v^0} a_k \dfrac{I_k^v}{\sum _{l \in \Omega _h} p_{lk} H_l} p_{mk} \left( H_m - I_m^h \right) \\ a_1 \dfrac{\sum _{i \in \Omega _h} c_i p_{i1} I_i^h}{\sum _{l \in \Omega _h} p_{l1} H_l} \left( V_1^* - I_1^v \right) \\ \vdots \\ a_n \dfrac{\sum _{i \in \Omega _h} c_i p_{in} I_i^h}{\sum _{l \in \Omega _h} p_{ln} H_l} \left( V_n^* - I_n^v \right) \end{pmatrix} \end{aligned} \\{} & {} \textrm{and } \ \begin{aligned} \mathscr {V}&= \begin{pmatrix} \gamma _1 I_1^h \\ \vdots \\ \gamma _m I_m^h \\ \mu _1 I_1^v - \sum \limits _{r \in \Omega _v} d_{1r} I_r^v \\ \vdots \\ \mu _n I_n^v - \sum \limits _{r \in \Omega _v} d_{nr} I_r^v \end{pmatrix}, \end{aligned} \end{aligned}$$

respectively. Evaluating the Jacobian matrices of $\mathscr {F}$ and $\mathscr {V}$ at $E_0$ gives the new infection and transition matrices as follows

$$\begin{aligned} \begin{aligned} F=\begin{pmatrix} 0_{m \times m} &{} \mathscr {A} \\ \mathscr {B} &{} 0_{n \times n} \end{pmatrix} \quad \textrm{and} \quad V= \begin{pmatrix} \mathscr {C} &{} 0_{m \times n} \\ 0_{n \times m} &{} \mathscr {D} \end{pmatrix}, \end{aligned} \end{aligned}$$

where

3.1 $$\begin{aligned} \begin{aligned} \mathscr {A}&=(a_{ij})_{m \times n}=\left( \frac{a_j b_i p_{ij} H_i }{\sum _{l \in \Omega _h} p_{lj} H_l} \right) _{m \times n},\\ \mathscr {B}&=(b_{ji})_{n \times m}=\left( \frac{a_j c_i p_{ij} V_j^* }{\sum _{l \in \Omega _h} p_{lj} H_l} \right) _{n \times m},\\ \mathscr {C}&=\textrm{diag}\left( \gamma _1,\cdots ,\gamma _m \right) ,\\ \mathscr {D}&=\textrm{diag}\left( \mu _1,\cdots ,\mu _n \right) -D. \end{aligned} \end{aligned}$$

Note that $a_{ij}=b_{ji}=0$ whenever $p_{ij}=0$ regardless of whether $j\in \Omega _v^0$ or not, and $\mathscr {D}^{-1}$ exists and is positive (see e.g., Lemma 1 in Gao et al. (2019)). The next generation matrix is

$$\begin{aligned} \begin{aligned} FV^{-1}=\begin{pmatrix} 0_{m \times m} &{} \mathscr {A} \mathscr {D}^{-1} \\ \mathscr {B}\mathscr {C}^{-1} &{} 0_{n \times n} \end{pmatrix}. \end{aligned} \end{aligned}$$

Thus, the basic reproduction number of model (2.3) is defined as

3.2 $$\begin{aligned} \begin{aligned} \mathcal {R}_0 = \rho (F V^{-1})=\sqrt{\rho (\mathscr {A} \mathscr {D}^{-1} \mathscr {B} \mathscr {C}^{-1})}=\sqrt{\rho ( \mathscr {B} \mathscr {C}^{-1}\mathscr {A} \mathscr {D}^{-1})}, \end{aligned} \end{aligned}$$

where $\rho$ represents the spectral radius of a square matrix, $\mathscr {A} \mathscr {D}^{-1} \mathscr {B} \mathscr {C}^{-1}$ is a square matrix of order *m*, and $\mathscr {B} \mathscr {C}^{-1}\mathscr {A} \mathscr {D}^{-1}$ is a square matrix of order *n*.

### Global dynamics

In this subsection, we will apply the theory of monotone dynamical systems (Smith 1995; Zhao 2017) to show the global dynamical behavior of the model system.

#### Theorem 3.1

Suppose ($\mathcal {H}1$) is valid for model (2.3). If $\mathcal {R}_0 \le 1$, then the disease-free equilibrium $E_0$ is globally asymptotically stable among nonnegative solutions; otherwise, there is a unique endemic equilibrium, denoted by $E^* =(I_1^{h*},\dots ,I_m^{h*},I_1^{v*},\dots ,I_n^{v *})$, which is globally asymptotically stable among positive solutions.

#### Proof

Denote the vector field associated to (2.3) by $\varvec{f}=(f_1,\dots ,f_{m+n})$. We rewrite model (2.3) in vector form $\varvec{x}'=\varvec{f}( \varvec{x} )$, where

$$\begin{aligned} \varvec{x}=\left( x_1,\dots ,x_m,x_{m+1},\dots ,x_{m+n} \right) =( I_1^h,\dots ,I_m^h,I_1^v,\dots ,I_n^v). \end{aligned}$$

Next we prove the global result by verifying the three conditions of Corollary 3.2 in Zhao and Jing (1996) or Theorem 2.3.4 in Zhao (2017) on the positively invariant set $\Gamma$.

1. The Jacobian matrix of $\varvec{f}$ at $\varvec{x}$ is
  $$\begin{aligned} D\varvec{f}(\varvec{x})=\left( \frac{\partial f_{s}}{\partial x_{r}}\right) _{(m+n)\times (m+n)}, \end{aligned}$$
  where
  $$\begin{aligned} \begin{aligned} \frac{\partial f_{s}}{\partial x_{r}}=\left\{ \begin{array}{ll} -b_s \sum \limits _{k \in \Omega _v^0} a_k \dfrac{I_k^v}{\sum _{l \in \Omega _h} p_{lk}H_l} p_{sk} - \gamma _s,&{} 1\le s=r\le m, \\ 0, &{} 1\le s,\, r\le m,\; s\ne r, \\ b_s \dfrac{a_{r-m} p_{s,r-m}}{\sum _{l \in \Omega _h} p_{l,r-m}H_{l}}\left( H_s-I_s^h \right) , &{} 1\le s\le m,\;m+1\le r\le m+n, \\ a_{s-m} \dfrac{c_r p_{r,s-m}}{\sum _{l\in \Omega _h}p_{l,s-m}H_l} \left( V_{s-m}^*-I_{s-m}^v \right) , &{} m+1\le s\le m+n,\;1\le r\le m, \\ -a_{s-m} \dfrac{\sum \limits _{i\in \Omega _{h}} c_i p_{i,s-m} I_i^h}{\sum \limits _{l\in \Omega _{h}}p_{l,s-m} H_l}-\mu _{s-m} +d_{s-m,s-m}, &{} m+1\le s=r\le m+n, \\ d_{s-m,r-m}, &{} m+1\le s,\, r\le m+n,\; s\ne r. \end{array}\right. \end{aligned} \end{aligned}$$
  Obviously, the matrix $D\varvec{f}(\varvec{x})$ is essentially nonnegative in the positively invariant set $\Gamma$. Therefore, system (2.3) is cooperative. Since the travel rates matrix of vectors $D=(d_{ij})_{n \times n}$ is irreducible and the residence times matrix of hosts *P* is nonnegative with row sum one, the matrix $F-V =D\varvec{f}(\varvec{0})$ is irreducible. In fact, the irreducibility of $F-V$ is equivalent to that of $$\left( {\begin{matrix}0_{m\times m}&{}P\\ P^{\textrm{T}}&{}D\end{matrix}}\right) $$. By Theorem 3.6 in Gao and Cao (2024), the host-vector network generated by model (2.3) is strongly connected. Therefore, $D\varvec{f}(\varvec{x})$ is irreducible in the interior of $\Gamma$, denoted by $\mathring{\Gamma }$. Furthermore, any nonzero solution starting from the boundary of $\Gamma$ will immediately enter and remain in $\mathring{\Gamma }$.
2. The origin is the disease-free equilibrium, i.e., $\varvec{f}(\varvec{0})=\varvec{0}$. In the proof of Proposition 2.1, we have showed $f_i(\varvec{x})\ge 0$ for all $\varvec{x}\in \Gamma$ with $x_i=0$, $1\le i\le m+n$; $f_i(\varvec{x})=-\gamma _i H_i<0$ for all $\varvec{x}\in \Gamma$ with $x_i=H_i$, $1\le i\le m$; $f_i(\varvec{x})\le -\mu _{i-m} V_{i-m}^*<0$ for all $\varvec{x}\in \Gamma$ with $x_i=V_{i-m}^*$, $m+1\le i\le m+n$.
3. For any $\varvec{x}\in \Gamma$ with $\varvec{x}\gg \varvec{0}$ and $\xi \in (0,1)$, we have
  $$\begin{aligned} \begin{aligned} f_{i}(\xi \varvec{x})-\xi f_{i}(\varvec{x}) =\xi (1-\xi ) b_i \sum _{k \in \Omega _v^0} a_k \frac{I_k^v}{\sum _{l \in \Omega _h}p_{lk}H_l} p_{ik} I_i^h >0, \quad i\in \Omega _h, \end{aligned} \end{aligned}$$
  and
  $$\begin{aligned} \begin{aligned} f_{m+j}(\xi \varvec{x})-\xi f_{m+j}(\varvec{x})= \left\{ \begin{array}{ll} \xi (1-\xi ) a_j \frac{\sum _{i \in \Omega _h}c_ip_{ij} I_i^h}{\sum _{l \in \Omega _h}p_{lj}H_l} I_j^v > 0,&{} \quad j\in \Omega _v^0,\\ 0, &{} \quad j\in \Omega _v\backslash \Omega _v^0. \end{array}\right. \end{aligned} \end{aligned}$$
  Therefore, $\varvec{f}$ is strongly subhomogeneous in $\Gamma$.

By Theorem 2 in van den Driessche and Watmough (2002) and Corollary 3.2 in Zhao and Jing (1996), if $s(D\varvec{f}(\varvec{0}))=s(F-V)\le 0$, i.e., $\mathcal {R}_0 \le 1$, then $E_0=\varvec{0}$ is globally asymptotically stable; if $s(D\varvec{f}(\varvec{0})) > 0$, i.e., $\mathcal {R}_0 > 1$, then the model system (2.3) admits a unique positive equilibrium $E^*$ which is globally attractive. Following an argument analogous to the proof of Theorem 3.1 or Remark 3.4 in Gao and Ruan (2011) or Theorem 3.2 in Zhao and Jing (1996) or Lemma 2.1 in Wu and Zhao (2022), $E^*$ is globally asymptotically stable. $\square$

### Disconnected patch model

We consider disease transmission within one patch and the associated single-patch basic reproduction number can be used to estimate the multi-patch basic reproduction number. We say patch $k\in \Omega _v^0$ is disconnected if there is no host or vector movement from/to patch *k*, and the distributions of hosts and vectors on the patch are the same as they are at the disease-free equilibrium. Namely, on patch *k* there are $V_k^*$ vectors and $\sum _{i\in \Omega _h}p_{ik}H_i$ hosts of which $p_{ik}H_i$ belong to group $i\in \Omega _h$. Let $I_{ik}^h$ denote the number of infected hosts of group *i* on patch *k*, and $I_k^v$ denote the number of infected vectors on patch *k*, the same as in connected case. The disease dynamics on disconnected patch $k\in \Omega _v^0$ are described by the following system of ordinary differential equations

3.3 $$\begin{aligned} \begin{aligned} \frac{dI_{ik}^h}{dt}&=a_k b_i \dfrac{ I_k^v }{\sum _{l \in \Omega _h} p_{lk} H_l} \left( p_{ik} H_i- I_{ik}^h \right) -\gamma _i I_{ik}^h, \quad i \in \Omega _h,\\ \frac{dI_k^v}{dt}&=a_k \dfrac{\sum _{i \in \Omega _h} c_i I_{ik}^h }{\sum _{l \in \Omega _h} p_{lk} H_l} \left( V_k^* - I_k^v \right) - \mu _k I_k^v. \end{aligned} \end{aligned}$$

Clearly, the origin is the disease-free equilibrium of model (3.3). The next generation matrix is given by

$$\begin{aligned} \begin{aligned} F_k V_k^{-1}&=\begin{pmatrix} 0_{m \times m} &{} \mathscr {A}_k \\ \mathscr {B}_k &{} 0 \end{pmatrix} \begin{pmatrix} \mathscr {C}_k &{} 0_{m \times 1} \\ 0_{1 \times m} &{} \mathscr {D}_k \end{pmatrix}^{-1} = \begin{pmatrix} 0_{m \times m} &{} \mathscr {A}_k \mathscr {D}_k^{-1} \\ \mathscr {B}_k \mathscr {C}_k^{-1} &{} 0 \end{pmatrix}, \end{aligned} \end{aligned}$$

where

$$\begin{aligned}&\mathscr {A}_k=\dfrac{a_k}{\sum _{l \in \Omega _h} p_{lk} H_l} \begin{pmatrix} b_1 p_{1k} H_1 \\ \vdots \\ b_m p_{mk} H_m \end{pmatrix},\qquad{} & {} \mathscr {B}_{k}=\dfrac{a_k V_k^*}{\sum _{l\in \Omega _h} p_{lk} H_l} (c_1, \dots , c_m), \\&\mathscr {C}_{k}=\textrm{diag}(\gamma _1,\dots ,\gamma _m),\qquad{} & {} \mathscr {D}_k=(\mu _k). \end{aligned}$$

The basic reproduction number of patch *k* in disconnection is

3.4 $$\begin{aligned} \begin{aligned} \mathcal {R}_0^{(k)}=&\rho (F_kV_k^{-1})=\sqrt{\rho (\mathscr {B}_k \mathscr {C}_k^{-1}\mathscr {A}_k \mathscr {D}_k^{-1})}\\ =&\sqrt{\frac{a_k^2 V_k^*}{\mu _k ( \sum _{l \in \Omega _h} p_{lk} H_l)^2 } \sum \limits _{i \in \Omega _h} \dfrac{b_i c_i p_{ik} H_i}{\gamma _i }}. \end{aligned} \end{aligned}$$

This can also be derived from the epidemiological perspective. The introduction of an infected vector into a completely susceptible patch *k* with $\sum _{i\in \Omega _h}p_{ik}H_i$ hosts and $V_k^*$ vectors will infect a number of

$$\begin{aligned} a_k\cdot \dfrac{p_{ik} H_i}{\sum _{l\in \Omega _h} p_{lk} H_l}\cdot \frac{1}{\mu _k}\cdot b_i \end{aligned}$$

hosts of group $i\in \Omega _h$. On the other hand, an infected host of group *i* can infect

$$\begin{aligned} \dfrac{a_k V_k^*}{\sum _{l\in \Omega _h} p_{lk} H_l}\cdot \frac{1}{\gamma _i}\cdot c_i \end{aligned}$$

vectors on patch *k*. Thus, the number of infected vectors produced by the introduction of one infected vector into disconnected patch *k* is

$$\begin{aligned} \sum _{i\in \Omega _h}\left( a_k\cdot \dfrac{p_{ik} H_i}{\sum _{l\in \Omega _h} p_{lk} H_l} \cdot \frac{1}{\mu _k}\cdot b_i\cdot \dfrac{a_k V_k^*}{\sum _{l\in \Omega _h} p_{lk} H_l}\cdot \frac{1}{\gamma _i}\cdot c_i\right) =\left( \mathcal {R}_0^{(k)}\right) ^2. \end{aligned}$$

In case of $k \in \Omega _v\backslash \Omega _v^0$, that is, patch *k* is host-free, it is natural to set $\mathcal {R}_0^{(k)}=0$. Following Theorem 3.1, the global dynamics of model (3.3) are also completely determined by its basic reproduction number.

#### Corollary 3.2

For model (3.3), the disease-free equilibrium is globally asymptotically stable if $\mathcal {R}_0^{(k)}\le 1$, and there exists a unique endemic equilibrium which is globally asymptotically stable if $\mathcal {R}_0^{(k)}>1$.

## Estimation and minimization of $\mathcal {R}_0$

Since the dynamic behavior of model (2.3) is governed by its basic reproduction number $\mathcal {R}_0$, it is desirable to eradicate a disease by reducing $\mathcal {R}_0$ to less than unity. Thus, we are interested in exploring the relationship between the basic reproduction number and its involved parameters. In particular, how does movement affect $\mathcal {R}_0$? This is generally a challenging question. We will focus on establishing bounds on $\mathcal {R}_0$ that are independent or dependent of movement-related parameters to measure the extent of host visitation and/or vector migration on disease persistence. In addition, we explore how to optimize vector control strategy in terms of $\mathcal {R}_0$.

### Movement-independent estimation of $\mathcal {R}_0$

We aim to find bounds of $\mathcal {R}_0$ of model (2.3) that are independent of the residence times matrix of hosts and the migration matrix of vectors. These suggest that the movement of hosts and vectors cannot infinitely increase or decrease the risk of infection.

The total numbers of hosts over all groups and vectors across all patches are denoted by

$$\begin{aligned} H=\sum _{i \in \Omega _h}H_i=\sum _{i \in \Omega _h}H_i(0) \quad \textrm{and } \quad V=\sum _{j \in \Omega _v}V_j^*=\sum _{j \in \Omega _v}V_j(0), \end{aligned}$$

respectively. Host group $i \in \Omega _h$ and vector patch $j\in \Omega _v$ account for

$$\begin{aligned} h_i=\frac{H_i}{H} \quad \textrm{and } \quad v^*_j=\dfrac{V^*_j}{V} \end{aligned}$$

of the total host and vector population sizes, respectively. The effective host size of patch $j \in \Omega _v$ is $\sum _{l \in \Omega _h} p_{lj} H_l$ which accounts for a proportion of $h_j ^*=\sum _{l\in \Omega _h} p_{lj}h_l$ of the total host population. Denote

$$\begin{aligned} \begin{aligned} \mathcal {R}_{ijkr}=&\sqrt{ \dfrac{a_i a_j b_k c_k V }{\gamma _k \mu _r H } }=\sqrt{a_i \cdot a_j \cdot \dfrac{b_k c_k}{ \gamma _k } \cdot \dfrac{1}{\mu _r} \cdot \dfrac{V}{H} },\qquad i,\,j,\,r \in \Omega _v,\ k \in \Omega _h. \end{aligned} \end{aligned}$$

The following lemma will be used to show the lower bound of $\mathcal {R}_0$ in terms of $\mathcal {R}_{ijkr}$. It is a simple generalization of Lemma 3 in Gao et al. (2019). We omit the proof which relies on a theorem on line-sum symmetric matrices (Eaves et al. 1985). Interested readers can complete the proof with a minor modification of that of Lemma 3 in Gao et al. (2019).

#### Lemma 4.1

Let $A=(a_{ij})_{n\times n}$ be an irreducible and essentially nonnegative matrix with zero column sums; $\varvec{w}=(w_1,\dots ,w_n)^{\textrm{T}}$ be the right positive eigenvector of *A* associated with the zero eigenvalue; $M=\textrm{diag}(w_1,\dots ,w_n)$ and $N=\textrm{diag}(r_1,\dots ,r_n)-A$ with $r_i>0$ for $1\le i\le n$. Assume $\varvec{x}=(x_1,\dots ,x_n)^{\textrm{T}}$ and $\varvec{y}=(y_1,\dots ,y_n)^{\textrm{T}}$ are two positive vectors, and $\lambda$ is a positive constant. Then if $N^{-1}M\varvec{x}=\lambda \varvec{y}$ then $\lambda \ge \sum _{i=1}^n\frac{x_i}{y_i}w_i^2/\sum _{i=1}^n r_iw_i$ with equality if and only if

$$\begin{aligned} \frac{y_1}{w_1}=\cdots =\frac{y_n}{w_n}\quad \text{ and } \quad \frac{x_1w_1}{y_1r_1}=\cdots =\frac{x_nw_n}{y_nr_n}=\lambda . \end{aligned}$$

#### Theorem 4.2

For model (2.3), if $\Omega _v^0=\Omega _v$, i.e., no patch is host-free, then

$$\begin{aligned} \mathcal {R}_0 \ge \min _{\begin{array}{c} k \in \Omega _h \\ i,\,j,\,r \in \Omega _v \end{array}} \mathcal {R}_{ijkr} = \min _{\begin{array}{c} k \in \Omega _h \\ i,\,j,\,r \in \Omega _v \end{array}} \sqrt{ \dfrac{a_i a_j b_k c_k V }{\gamma _k \mu _r H }}, \end{aligned}$$

i.e., the basic reproduction number $\mathcal {R}_0$ has a lower bound that is independent of the residence times matrix *P* of hosts and the migration matrix *D* of vectors.

#### Proof

The assumption $\Omega _v^0=\Omega _v$ implies that $h_j^*=\sum _{l\in \Omega _h} p_{lj}H_l/H=\sum _{l\in \Omega _h} p_{lj}h_l>0$ for all $j\in \Omega _v$, and $\varvec{1}P^{\textrm{T}}P\gg \varvec{0}$ and $P^{\textrm{T}}P\varvec{1}^{\textrm{T}}\gg \varvec{0}$ with $\varvec{1}=(1,\dots ,1)$. Following (3.2), the basic reproduction number of model (2.3) is

$$\begin{aligned} \mathcal {R}_0=\sqrt{\rho (\mathscr {B} \mathscr {C}^{-1}\mathscr {A} \mathscr {D}^{-1})}= \sqrt{\rho (\mathscr {D}^{-1}\mathscr {B} \mathscr {C}^{-1}\mathscr {A})}=\sqrt{\rho (\mathscr {D}^{-1}(\beta _{ij})_{n \times n})}, \end{aligned}$$

where $$(\beta _{ij})_{n \times n}=\mathscr {B} \mathscr {C}^{-1}\mathscr {A}$$ and

4.1 $$\begin{aligned} \beta _{ij}&=\sum _{k \in \Omega _h} \left( \frac{a_i a_j b_k c_k}{\gamma _k}\cdot \dfrac{ p_{ki} V_i^* }{\sum _{l \in \Omega _h } p_{li} H_l } \cdot \dfrac{p_{kj} H_k }{ \sum _{l \in \Omega _h} p_{lj} H_l } \right) \end{aligned}$$

4.2 $$\begin{aligned}&=\sum _{k \in \Omega _h} \left( \frac{a_i a_j b_k c_k V}{\gamma _k H}\cdot \dfrac{ p_{ki} v_i^* }{h_i^* } \cdot \dfrac{p_{kj} h_k }{ h_j^* } \right) . \end{aligned}$$

Define

$$\begin{aligned} \begin{aligned} L=(l_{ij})_{n \times n}= \left( \sum _{k \in \Omega _h} \left( \dfrac{ p_{ki} v_i^* }{h_i^* } \cdot \dfrac{p_{kj} h_k }{ h_j^* } \right) \right) _{n \times n}\ \text{ and } \ \hat{L}=\mathscr {D}^{-1}L. \end{aligned} \end{aligned}$$

Clearly,

4.3 $$\begin{aligned} \begin{aligned} \min _{\begin{array}{c} k \in \Omega _h \\ i,j \in \Omega _v \end{array}} \frac{a_i a_j b_k c_k V }{\gamma _k H} \hat{L} \le \mathscr {D}^{-1}\mathscr {B} \mathscr {C}^{-1}\mathscr {A} \le \max _{\begin{array}{c} k \in \Omega _h \\ i,j \in \Omega _v \end{array}} \frac{a_i a_j b_k c_k V }{\gamma _k H} \hat{L}. \end{aligned} \end{aligned}$$

It suffices to show that $\rho (\hat{L})\ge \min \limits _{r\in \Omega _v}\frac{1}{\mu _r}$.

We rewrite

4.4 $$\begin{aligned} \begin{aligned} \hat{L}&= \mathscr {D}^{-1}\left( \frac{ p_{ki} v_i^* }{h_i^*}\right) _{n \times m} \left( \frac{ p_{kj} h_k }{h_j^*} \right) _{m \times n} =\mathscr {D}^{-1}\mathscr {B}_1 \mathscr {B}_2 \mathscr {A}_1, \end{aligned} \end{aligned}$$

where

$$\begin{aligned} \mathscr {B}_1=\textrm{diag}\left( v_1^*,\dots ,v_n^* \right) ,\quad \mathscr {B}_2= \left( \frac{ p_{ki} }{h_i^*} \right) _{n \times m},\quad \mathscr {A}_1=\left( \frac{ p_{kj} h_k }{h_j^*}\right) _{m \times n}. \end{aligned}$$

Since $\hat{L}$ is a positive matrix, by the Perron–Frobenius theorem (Horn and Johnson 2013), there exists a positive right eigenvector $\varvec{y}$ corresponding to the eigenvalue $\rho (\hat{L})$ such that

$$\begin{aligned} \begin{aligned} \mathscr {D}^{-1} \mathscr {B}_1 \mathscr {B}_2 \mathscr {A}_1 \varvec{y}=\mathscr {D}^{-1} \mathscr {B}_1\varvec{x}=\rho (\hat{L}) \varvec{y}. \end{aligned} \end{aligned}$$

Here $\varvec{x}:=\mathscr {B}_2 \mathscr {A}_1 \varvec{y} \gg \varvec{0}$. Applying Lemma 4.1, we have

$$\begin{aligned} \begin{aligned} \rho (\hat{L})&\ge \sum _{i \in \Omega _v}\frac{x_i}{y_i}(v_i^*)^2\Big /\sum _{i \in \Omega _v} \mu _iv_i^*\\&\ge \min _{r\in \Omega _v} \dfrac{1}{\mu _r^v} \sum _{i \in \Omega _v}\frac{x_i}{y_i}(v_i^*)^2 \Big /\sum _{i \in \Omega _v} v_i^* = \min _{r\in \Omega _v} \dfrac{1}{\mu _r^v} \sum _{i \in \Omega _v} \frac{ x_i}{y_i} (v_i^*)^2. \end{aligned} \end{aligned}$$

Direct calculation gives

$$\begin{aligned} \begin{aligned} \sum _{i \in \Omega _v} \frac{ x_i}{y_i} (v_i^*)^2&= \sum _{i \in \Omega _v} \left( \sum _{j \in \Omega _v} \sum _{k \in \Omega _h} \frac{p_{ki} p_{kj} h_k}{h_i^* h_j^*} y_j \right) \frac{1}{y_i} (v_i^*)^2\\&= \sum _{k \in \Omega _h} h_k \left( \sum _{i \in \Omega _v} \frac{p_{ki}(v_i^*)^2}{h_i^* y_i } \right) \left( \sum _{j \in \Omega _v} \frac{p_{kj}y_j}{h_j^*} \right) \\&\ge \sum _{k \in \Omega _h} h_k \left( \sum _{i \in \Omega _v} \frac{p_{ki}v_i^*}{h_i^*} \right) ^2, \end{aligned} \end{aligned}$$

by the Cauchy–Schwarz inequality. Note that $\sum _{k \in \Omega _h}h_k=1$, then again by the Cauchy–Schwarz inequality,

$$\begin{aligned} \begin{aligned} \sum _{i \in \Omega _v} \frac{ x_i}{y_i} (v_i^*)^2&\ge \sum _{k \in \Omega _h} h_k \left( \sum _{i \in \Omega _v} \frac{p_{ki}v_i^*}{h_i^*} \right) ^2 \cdot \sum _{k \in \Omega _h}h_k\\&\ge \left( \sum _{k \in \Omega _h} h_k \sum _{i \in \Omega _v} \frac{p_{ki}v_i^*}{h_i^*} \right) ^2 = \left( \sum _{i \in \Omega _v} \sum _{k \in \Omega _h} \frac{p_{ki} v_i^*h_k }{h_i^*} \right) ^2\\&= \left( \sum _{i \in \Omega _v} v_i^* \frac{\sum _{k \in \Omega _h}p_{ki}h_k}{h_i^*} \right) ^2 = \left( \sum _{i \in \Omega _v} v_i^* \right) ^2 =1. \end{aligned} \end{aligned}$$

Thus,

$$\begin{aligned} \rho ( \hat{L} ) \ge \min _{r\in \Omega _v} \dfrac{1}{\mu _r} \sum _{i \in \Omega _v} \frac{ x_i}{y_i} (v_i^*)^2 \ge \min _{r\in \Omega _v} \dfrac{1}{\mu _r}. \end{aligned}$$

It follows from (4.3) that

$$\begin{aligned} \mathcal {R}_0^2= & {} \rho ( \mathscr {D}^{-1} \mathscr {B} \mathscr {C}^{-1}\mathscr {A} ) \ge \min _{\begin{array}{c} k \in \Omega _h \\ i,j \in \Omega _v \end{array}} \dfrac{a_i a_j b_k c_k V }{\gamma _k H} \rho ( \hat{L} ) \\\ge & {} \min _{\begin{array}{c} k \in \Omega _h \\ i,\,j,\,r \in \Omega _v \end{array}} \dfrac{a_i a_j b_k c_k V }{\gamma _k \mu _r H} \ge \min _{\begin{array}{c} k \in \Omega _h \\ i,\,j,\,r \in \Omega _v \end{array}} \mathcal {R}_{ijkr}^2. \end{aligned}$$

This completes the proof. $\square$

Next, we estimate the upper bound of $\mathcal {R}_0$ in terms of

$$\begin{aligned} \begin{aligned} \mathcal {R}_{ijkj}=&\sqrt{ \dfrac{a_i a_j b_k c_k V }{\mu _j\gamma _k H } }=\sqrt{a_i \cdot \dfrac{a_j}{\mu _j} \cdot \dfrac{b_k c_k}{ \gamma _k } \cdot \dfrac{ V}{H}}, \qquad i,\,j \in \Omega _v, \; k \in \Omega _h. \end{aligned} \end{aligned}$$

Here $\mathcal {R}_{ijkj}$ is a reproduction number that involves vector patches *i* and *j* and host group *k*. In fact, introducing an infected vector to patch *j* can infect $a_j \cdot \frac{1}{\mu _j} \cdot b_k$ susceptible hosts of group *k* and an infected host of group *k* can infect $\frac{a_i V}{H} \cdot \frac{1}{\gamma _k} \cdot c_k$ susceptible vectors in patch *i*. Their product is just $\mathcal {R}_{ijkj}^2$.

#### Theorem 4.3

For model (2.3), the basic reproduction number $\mathcal {R}_0$ has an upper bound that is independent of the residence times matrix *P* of hosts and the migration matrix *D* of vectors, namely,

$$\begin{aligned} \mathcal {R}_0 \le \max \limits _{ l \in \Omega _h}\frac{1}{\sqrt{h_l}}\cdot \max _{\begin{array}{c} k \in \Omega _h \\ i,\, j \in \Omega _v \end{array}} \mathcal {R}_{ijkj}. \end{aligned}$$

#### Proof

It follows from (4.2) that

$$\begin{aligned} \mathscr {D}^{-1}\mathscr {B} \mathscr {C}^{-1}\mathscr {A}\le \max _{\begin{array}{c} k \in \Omega _h \\ i \in \Omega _v \end{array}} \frac{a_i b_k c_k V }{\gamma _k H} \hat{L}_1, \end{aligned}$$

where

$$\begin{aligned} \begin{aligned} \hat{L}_1=\mathscr {D}^{-1}\left( \sum _{k \in \Omega _h} \left( \dfrac{ p_{ki} v_i^* }{h_i^* } \cdot \dfrac{a_j p_{kj} h_k }{ h_j^* } \right) \right) _{n \times n}= \mathscr {D}^{-1}\hat{\mathscr {B}} \mathscr {A}_1 \mathscr {A}_2, \end{aligned} \end{aligned}$$

and

$$\begin{aligned} \begin{aligned} \hat{\mathscr {B}}= \mathscr {B}_1 \mathscr {B}_2 = \left( \frac{ p_{ki} v_i^* }{h_i^*}\right) _{n \times m}, \quad \mathscr {A}_1=\left( \frac{ p_{kj} h_k }{h_j^*} \right) _{m \times n}, \quad \mathscr {A}_2= \textrm{diag}(a_1,\dots ,a_n). \end{aligned} \end{aligned}$$

Thus, it suffices to show that

$$\begin{aligned} \begin{aligned} \rho ( \hat{L}_1) =\rho (\mathscr {D}^{-1} \hat{\mathscr {B}}\mathscr {A}_1\mathscr {A}_2 )\le \max \limits _{ l \in \Omega _h}\frac{1}{h_l}\cdot \max \limits _{j\in \Omega _v}\frac{a_j}{\mu _j}. \end{aligned} \end{aligned}$$

Denote $U=\textrm{diag}(\mu _1,\dots ,\mu _n)$. A similarity transformation for matrix $\hat{L}_1$ gives

$$\begin{aligned} E=\left( e_{ij} \right) _{n \times n}=U\mathscr {D}^{-1} \hat{\mathscr {B}}\mathscr {A}_1\mathscr {A}_2U^{-1} \end{aligned}$$

satisfying $\rho (E)=\rho ( \hat{L}_1)$. Since *E* is a nonnegative matrix, by Theorem 8.1.22 in Horn and Johnson (2013), we can get

$$\begin{aligned} \rho (E) \le \max _{j\in \Omega _v} \sum _{i\in \Omega _v} e_{ij}. \end{aligned}$$

Note that $\varvec{1}\mathscr {D}=\varvec{1}U\Leftrightarrow \varvec{1}U\mathscr {D}^{-1}=\varvec{1}$, so

$$\begin{aligned} \begin{aligned} {\textbf {1}} E&= {\textbf {1}} U\mathscr {D}^{-1} \hat{\mathscr {B}} \mathscr {A}_1 \mathscr {A}_2 U^{-1} = {\textbf {1}}\hat{\mathscr {B}} \mathscr {A}_1 \mathscr {A}_2 U^{-1}\\&= (1,\dots ,1) \left( \dfrac{ p_{ki} v_i^* }{h_i^*}\right) _{n \times m}\mathscr {A}_1 \mathscr {A}_2 U^{-1}= \left( \sum _{i \in \Omega _v^0} \dfrac{ p_{ki} v_i^* }{h_i^*} \right) _{1 \times m}\mathscr {A}_1 \mathscr {A}_2 U^{-1}. \end{aligned} \end{aligned}$$

Let $\Omega _v^k=\{i\in \Omega _v^0 \mid p_{ki}>0\}$ be the set of patches visited by hosts of group *k*. Then

$$\begin{aligned} \begin{aligned} \sum _{i \in \Omega _v^0} \dfrac{ p_{ki} v_i^* }{h_i^*} = \sum _{i \in \Omega _v^k} \dfrac{ p_{ki} v_i^* }{\sum _{l \in \Omega _h} p_{li} h_l} \le \sum _{i \in \Omega _v^k} \frac{ p_{ki} v_i^* }{ p_{ki} h_k} = \sum _{i \in \Omega _v^k} \frac{ v^*_i }{ h_k} = \frac{ \sum _{i \in \Omega _v^k} v^*_i }{ h_k} \le \frac{1}{h_k}, \end{aligned} \end{aligned}$$

which implies that

$$\begin{aligned} \begin{aligned} \varvec{1} E&\le \left( \frac{1}{h_k}\right) _{1 \times m} \mathscr {A}_1 \mathscr {A}_2 U^{-1} \le \max _{k \in \Omega _h } \frac{1}{h_k} {\textbf {1}} \mathscr {A}_1 \mathscr {A}_2 U^{-1} \\&= \max _{k \in \Omega _h } \frac{1}{h_k} (1,\dots , 1)_{1 \times m} \left( \frac{ p_{kj} h_k }{h_j^*} \right) _{m \times n} \mathscr {A}_2 U^{-1} \\&= \max _{k \in \Omega _h } \frac{1}{h_k} \left( \sum _{k \in \Omega _h} \frac{ p_{kj} h_k }{h_j^*} \right) _{1 \times n} \mathscr {A}_2 U^{-1} \\&= \max _{k \in \Omega _h } \frac{1}{h_k} (1,\dots ,1)_{1 \times n} \mathscr {A}_2 U^{-1}=\max _{k \in \Omega _h } \frac{1}{h_k}\left( \frac{a_1}{\mu _1},\dots ,\frac{a_n}{\mu _n}\right) . \end{aligned} \end{aligned}$$

Therefore,

$$\begin{aligned} \rho (E) \le \max _{k \in \Omega _h } \frac{1}{h_k}\cdot \max _{j \in \Omega _v } \frac{a_j}{\mu _j}. \end{aligned}$$

The proof is completed by noting that $\rho ( \hat{L}_1)=\rho (E)$. $\square$

The above analysis provides lower and upper bounds of the basic reproduction number $\mathcal {R}_0$ for model (2.3) independent of host and vector movement in a heterogeneous environment. These especially hold for a homogeneous environment where all host groups and vector patches have the same epidemiological and demographic features, namely

$$\begin{aligned} a_j=a,\ b_i=b,\ c_i=c,\ \gamma _i=\gamma , \ \mu _j=\mu ,\quad \forall \ i \in \Omega _h, \ \forall \ j \in \Omega _v. \end{aligned}$$

#### Corollary 4.4

Let $\mathcal {R}_0(m/n)$ be the basic reproduction number of model (2.3) in a homogeneous environment with *m* host groups and *n* vector patches. Then $\mathcal {R}_0(m/n)$ has an upper bound independent of the residence times matrix *P* and the migration matrix *D*, i.e.,

$$\begin{aligned} \mathcal {R}_0(m/n) \le \max _{l \in \Omega _h}{\frac{1}{\sqrt{h_l}}} \cdot \mathcal {R}_0(1/1)=\max _{l \in \Omega _h}{\frac{1}{\sqrt{h_l}}}\cdot \sqrt{\frac{a^2 bcV}{ \mu \gamma H}}. \end{aligned}$$

If $\Omega _v^0=\Omega _v$, then $\mathcal {R}_0(m/n)$ has a lower bound independent of the residence times matrix *P* and the migration matrix *D*, i.e.,

$$\begin{aligned} \mathcal {R}_0(m/n) \ge \mathcal {R}_0(1/n)=\mathcal {R}_0(m/1)=\mathcal {R}_0(1/1)=\sqrt{\frac{a^2 bcV}{ \mu \gamma H}} \end{aligned}$$

with the equality holds if $h_i^*=v_i^*$ for all $i \in \Omega _v$, i.e., the distributions of hosts and vectors are proportional across all patches.

#### Proof

The lower and upper bounds of $\mathcal {R}_0(m/n)$ can be obtained directly from Theorems 4.2 and 4.3. It remains to verify the conditions under which the lower bound is reached. If $\Omega _v^0=\Omega _v$, then it follows from (4.2)–(4.4) that

$$\begin{aligned} \mathcal {R}_0(m/n)=\sqrt{\frac{a^2 b c V }{\gamma H}}\cdot \sqrt{ \rho ( \hat{L}) }, \end{aligned}$$

where $$\hat{L}=\mathscr {D}^{-1}\hat{\mathscr {B}} \mathscr {A}_1$$ with

$$\begin{aligned} \mathscr {D}=\mu {\mathbb {I}}-D,\quad \hat{\mathscr {B}}=\mathscr {B}_1\mathscr {B}_2= \left( \frac{ p_{ki} v_i^* }{h_i^*} \right) _{n \times m},\quad \mathscr {A}_1=\left( \frac{ p_{kj} h_k }{h_j^*}\right) _{m \times n}. \end{aligned}$$

The facts $\varvec{1}\mathscr {D}=\mu \varvec{1}$ and $\sum _{k \in \Omega _h} p_{kj} h_k=h_j^*,\, \forall \, j \in \Omega _v$ imply that $\varvec{1}\mathscr {D}^{-1}=\frac{1}{\mu }\varvec{1}$ and $\varvec{1}\mathscr {A}_1=\varvec{1}$, respectively. We claim that $\varvec{1}\hat{\mathscr {B}}=\varvec{1}$.

Case 1 If $h_i^*=v_i^*$, $\forall i\in \Omega _v$, then $\hat{\mathscr {B}}=P^{\textrm{T}}$ and hence $\varvec{1}\hat{\mathscr {B}}=\varvec{1}P^{\textrm{T}}=\varvec{1}$.

Case 2 If $m=1$, then $p_{1i}=h_i^*$ for all $i\in \Omega _v$ and hence

$$\begin{aligned} \varvec{1}\hat{\mathscr {B}}=\varvec{1}(v_i^*)_{n \times 1}=\sum \nolimits _{i\in \Omega _v}v_i^*=1. \end{aligned}$$

Case 3 If $n=1$, then $h_1^*=v_1^*=1$. By Case 1 or $p_{k1}=1$ for all $k\in \Omega _h$, it means

$$\begin{aligned} 1\hat{\mathscr {B}}=\left( \frac{ p_{k1} v_1^* }{h_1^*} \right) _{1 \times m}= (1)_{1 \times m}=\varvec{1}. \end{aligned}$$

In all three cases, we have

$$\begin{aligned} \varvec{1}\hat{L}=\varvec{1}\mathscr {D}^{-1}\hat{\mathscr {B}} \mathscr {A}_1=\frac{1}{\mu }\varvec{1}\hat{\mathscr {B}} \mathscr {A}_1 =\frac{1}{\mu }\varvec{1}\mathscr {A}_1=\frac{1}{\mu }\varvec{1}. \end{aligned}$$

It follows from the Perron–Frobenius theorem that $\rho ( \hat{L})=\frac{1}{\mu }$. $\square$

#### Remark 4.5

The lower bound $\mathcal {R}_0(1/1)$ means that a nonhomogeneous mixing of hosts and vectors driven by host and/or vector movements increases the disease persistence in a homogenous environment. Similar result was first proved by Dye and Hasibeder (1986), Hasibeder and Dye (1988) for a Lagrangian vector-borne disease model with only vector movement. Together with the recent works of Gao et al. (2019) and Gao and Cao (2024), we show that the conclusion holds for host and/or vector movements with Lagrangian and/or Eulerian approaches.

#### Remark 4.6

The equality $\mathcal {R}_0(1/n)=\mathcal {R}_0(m/1)=\mathcal {R}_0(1/1)$ can be derived epidemiologically. In fact, if $m=1$, i.e., only one host group, then the introduction of an infected host will cause

$$\begin{aligned} p_{1k} \cdot \dfrac{a V_k^*}{p_{1k} H} \cdot \dfrac{1}{\gamma }\cdot c = \dfrac{ac}{\gamma H} V_k^* \end{aligned}$$

vector infections in patch $k \in \Omega _v$, while an infected vector of patch *k* will lead to

$$\begin{aligned} a \cdot \dfrac{1}{\mu } \cdot b=\dfrac{ab}{\mu } \end{aligned}$$

host infections. So, the basic reproduction number involving one host group and *n* vector patches is

$$\begin{aligned} \mathcal {R}_0(1/n)=\sqrt{\sum _{k \in \Omega _v} \left( \dfrac{ac}{\gamma H}V_k^* \cdot \dfrac{ab}{\mu }\right) } = \sqrt{\dfrac{a^2 bc V}{\gamma \mu H}}=\mathcal {R}_0(1/1). \end{aligned}$$

If $n=1$, i.e., only one vector patch, then the introduction of an infected vector into the patch will infect

$$\begin{aligned} a\cdot \frac{H_i}{H} \cdot \dfrac{1}{\mu } \cdot b=\dfrac{abH_i}{\mu H} \end{aligned}$$

hosts of group *i*, while an infected host of group *i* will cause

$$\begin{aligned} \frac{aV}{H}\cdot \frac{1}{\gamma }\cdot c=\frac{acV}{\gamma H} \end{aligned}$$

vector infections. Thus, the basic reproduction number involving *m* host groups and one vector patch is

$$\begin{aligned} \mathcal {R}_0(m/1)=\sqrt{\sum _{i \in \Omega _h} \left( \dfrac{abH_i}{\mu H} \cdot \frac{acV}{\gamma H}\right) }= \sqrt{\dfrac{a^2 bc V}{\gamma \mu H}}=\mathcal {R}_0(1/1). \end{aligned}$$

#### Remark 4.7

The lower bound of $\mathcal {R}_0(m/n)$ is theoretically achievable through a consistent distribution of hosts and vectors, i.e., $h_i^*=v_i^*$ for all $i\in \Omega _v$. Denote $\varvec{H}=(H_1,\dots ,H_m)\gg \varvec{0}$ and $\varvec{V}=(V_1(0),\dots ,V_n(0))\gg \varvec{0}$, the initial population sizes of host groups and vector patches, respectively. Suppose the migration matrix *D* for vectors is fixed, then the distribution of vectors at equilibrium is $\varvec{V}^*=(V_1^*,\dots ,V_n^*)=V(v_1^*,\dots ,v_n^*)$ which is determined by *D* and $V=\sum _{i\in \Omega _v}V_i(0)$. By Remark 4.5 in Gao and Cao (2024), for example, one can set the residence times matrix of hosts to $P_1=((1,\dots ,1)_{1\times m})^{\textrm{T}} \cdot (v_1^*,\dots ,v_n^*)$, then

$$\begin{aligned} \begin{aligned} \varvec{H}P_1&=\varvec{H}((1,\dots ,1)_{1\times m})^{\textrm{T}} \cdot (v_1^*,\dots ,v_n^*)=H(v_1^*,\dots ,v_n^*) \\&= \bigg (\sum _{l \in \Omega _h} p_{l1} H_l,\dots ,\sum _{l \in \Omega _h} p_{ln} H_l\bigg )= H(h_1^*,\dots ,h_n^*), \end{aligned} \end{aligned}$$

that is, $h_i^*=v_i^*$ for all $i\in \Omega _v$. On the other hand, suppose the residence times matrix *P* for hosts is fixed, then the distribution of effective host sizes is $H(h_1^*,\dots ,h_n^*)$ which is determined by *P* and $\varvec{H}$. Following Remark 2 in Gao et al. (2019), for example, the migration matrix of vectors can be set as $D_1=(h_1^*,\dots ,h_n^*)^{\textrm{T}}\cdot (1,\dots ,1)_{1\times n}-{\mathbb {I}}_n$, then

$$\begin{aligned} \begin{aligned} D_1(h_1^*,\dots ,h_n^*)^{\textrm{T}}&=((h_1^*,\dots ,h_n^*)^{\textrm{T}}\cdot (1,\dots ,1)_{1\times n}-{\mathbb {I}}_n)(h_1^*,\dots ,h_n^*)^{\textrm{T}} \\&=(h_1^*,\dots ,h_n^*)^{\textrm{T}}\cdot \sum \nolimits _{i\in \Omega _v}h_i^*-(h_1^*,\dots ,h_n^*)^{\textrm{T}}\\&=(h_1^*,\dots ,h_n^*)^{\textrm{T}}-(h_1^*,\dots ,h_n^*)^{\textrm{T}}=\varvec{0}. \end{aligned} \end{aligned}$$

By the Perron–Frobenius theorem, we know $h_i^*=v_i^*$ for all $i\in \Omega _v$.

#### Remark 4.8

Given a migration matrix *D*, the residence times matrix *P* such that the distributions of hosts and vectors are consistent is not unique. Indeed, in addition to

$$\begin{aligned} \varvec{H}P=H(v_1^*,\dots ,v_n^*),\ \text{ i.e. },\ \sum \nolimits _{l \in \Omega _h} p_{li} H_l=Hv_i^*,\ i\in \Omega _v, \end{aligned}$$

the matrix *P* has row sums equal one, i.e., $\sum _{i\in \Omega _v}p_{li}=1,\ \forall \, l\in \Omega _h$, and $\varvec{H}P\varvec{1}^{\textrm{T}}=H=H(v_1^*,\dots ,v_n^*)\varvec{1}^{\textrm{T}}$ always holds. So, the system of linear equations in terms of $p_{li}$, $l\in \Omega _h$ and $i\in \Omega _v$, is compatible (since $P_1$ is a solution) and at least has $mn-n-m+1=(m-1)(n-1)$ free variables. Let $\bar{P}$ be any solution of the corresponding homogeneous system of $p_{lj}$. Then, for small enough $\tau >0$, the matrix $P_1+\tau \bar{P}$ is a nonnegative solution to the inhomogeneous system of $p_{lj}$, namely a qualified residence times matrix. It is interesting but tedious to solve all the “basis” matrices. Any convex combination of these matrices still makes the distributions of hosts and vectors consistent.

It is worth pointing out that the lower bound $\mathcal {R}_0(1/1)$ can be reached even if $h_i^*=v_i^*$ does not hold for all $i\in \Omega _v$. For example, $\mathcal {R}_0(m/n)=\mathcal {R}_0(1/1)$ if the residence times matrix

$$\begin{aligned} P=P_2:=((1,\dots ,1)_{1\times m})^{\textrm{T}} \cdot (p_{11},\dots ,p_{1n}), \end{aligned}$$

i.e., $p_{li}=p_{ki}$, $\forall \, l, \, k \in \Omega _h, \, i \in \Omega _v$. Note that $P_2$ becomes $P_1$ when $p_{1i}=v_i^*$ for $i\in \Omega _v$. Actually, following the proof of Corollary 4.4, it suffices to check $\varvec{1}\hat{\mathscr {B}}=\varvec{1}$, which is true due to

$$\begin{aligned} \varvec{1}\hat{\mathscr {B}}= & {} \varvec{1}\left( \frac{ p_{ki} v_i^* }{h_i^*} \right) _{n \times m}=\varvec{1}\left( \frac{ p_{ki} v_i^* }{\sum _{l\in \Omega _h}p_{li}h_l} \right) _{n \times m}\\= & {} \varvec{1}\left( \frac{ p_{ki} v_i^* }{p_{ki}\sum _{l\in \Omega _h}h_l} \right) _{n \times m}=\varvec{1}(v_i^*)_{n \times m} =\varvec{1}. \end{aligned}$$

However, the distributions of hosts and vectors are inconsistent if

$$\begin{aligned} \varvec{H}P_2=\varvec{H}((1,\dots ,1)_{1\times m})^{\textrm{T}} \cdot (p_{11},\dots ,p_{1n})=H(p_{11},\dots ,p_{1n}) \ne H(v_1^*,\dots ,v_n^*). \end{aligned}$$

This result is not surprising since all rows of $P_2$ are the same, namely different host groups spend the same proportion of time on the same patch. In this case, there is no difference between host groups and hence the *m* groups can be combined into one group. Thus, we have $\mathcal {R}_0(m/n)=\mathcal {R}_0(1/n)=\mathcal {R}_0(1/1)$.

Interestingly, there are other scenarios where $\mathcal {R}_0(m/n)=\mathcal {R}_0(1/1)$. Consider a two-group and three-patch homogeneous environment with the setting:

$$\begin{aligned} (h_1,h_2)=\left( \frac{1}{2}, \frac{1}{2}\right) ,\ (v_1^*,v_2^*,v_3^*)=\left( \frac{1}{4},\frac{1}{3},\frac{5}{12}\right) ,\ \text{ and } \ P=\begin{pmatrix} \frac{1}{8} &{} \frac{1}{2} &{} \frac{3}{8} \\ \frac{1}{4} &{} \frac{1}{2} &{} \frac{1}{4} \end{pmatrix}. \end{aligned}$$

Direct calculation gives

$$\begin{aligned} (h_1^*,h_2^*,h_3^*)=(h_1,h_2)P=\left( \frac{3}{16},\frac{1}{2},\frac{5}{16}\right) \ne (v_1^*,v_2^*,v_3^*), \end{aligned}$$

but

$$\begin{aligned} \varvec{1}\hat{\mathscr {B}}=\varvec{1}\left( \frac{ p_{ki} v_i^* }{h_i^*} \right) _{3 \times 2}=\left( \frac{v_1^*}{h_1^*},\frac{v_2^*}{h_2^*},\frac{v_3^*}{h_3^*}\right) P^{\textrm{T}} =\left( \frac{4}{3},\frac{2}{3},\frac{4}{3}\right) P^{\textrm{T}}=\varvec{1}. \end{aligned}$$

#### Remark 4.9

When $\Omega _v^0 \ne \Omega _v$, i.e., at least one patch is host-free, the lower bound $\mathcal {R}_0(1/1)$ is no longer valid. Consider, for example, a homogeneous environment containing *m* host groups and two vector patches, where patch 2 is host-free, i.e., $p_{i1}=1$ and $p_{i2}=0,\, \forall \, i \in \Omega _h$. Then it follows from the proof of Corollary 4.4 and

$$\begin{aligned} \varvec{1}\hat{\mathscr {B}}=\varvec{1}\textrm{diag}\left( \frac{v_1^*}{h_1^*},0\right) P^{\textrm{T}}=\varvec{1} \begin{pmatrix} v_1^* &{} 0 \\ 0 &{} 0 \end{pmatrix}\begin{pmatrix} 1 &{} \cdots &{} 1\\ 0 &{} \cdots &{} 0 \end{pmatrix}=v_1^*\varvec{1} \end{aligned}$$

that $$\mathcal {R}_0(m/2)=\sqrt{v_1^*}\cdot \mathcal {R}_0(1/1)<\mathcal {R}_0(1/1)$$.

### Movement-dependent estimation of $\mathcal {R}_0$

In this subsection, we will obtain two kinds of estimates on the basic reproduction number: one depends on the vector movement in heterogeneous environment, and the other depends on both vector and host movements in homogeneous environment. Define

$$\begin{aligned} \begin{aligned} \tilde{\mathcal {R}}_{ijk}&=\sqrt{ \frac{a_i a_j b_k c_k V_i^* }{\mu _j \gamma _k H_k } }=\sqrt{a_iV_i^* \cdot \dfrac{a_j}{\mu _j} \cdot \dfrac{b_k c_k}{ \gamma _k H_k }}, \quad i,\,j \in \Omega _v, \,k \in \Omega _h, \end{aligned} \end{aligned}$$

which depends on the migration matrix of vectors and also involves vector patches *i* and *j* and host group *k*.

#### Theorem 4.10

The basic reproduction number $\mathcal {R}_0$ of model (2.3) has an upper bound that is independent of the host residence times matrix *P*, namely

$$\begin{aligned} \mathcal {R}_0 \le \sqrt{n} \max _{\begin{array}{c} k \in \Omega _h \\ i,\,j \in \Omega _v \end{array}} \tilde{\mathcal {R}}_{ijk}. \end{aligned}$$

#### Proof

It follows from (4.1) that

$$\begin{aligned} \beta _{ij}=\sum _{k \in \Omega _h} \left( \frac{a_i b_k c_k V_i^*}{\gamma _k H_k} \cdot \dfrac{ p_{ki}h_k }{h_i^* } \cdot \dfrac{ p_{kj} h_k }{h_j^* }\cdot a_j \right) . \end{aligned}$$

Denote

$$\begin{aligned} \check{L}=(\check{l}_{ij})_{n \times n}=\left( \sum _{k \in \Omega _h} \left( \dfrac{ p_{ki}h_k }{h_i^* } \cdot \dfrac{ p_{kj} h_k }{h_j^* } \right) \right) _{n \times n}\ \text{ and } \ \mathscr {A}_2= \textrm{diag}(a_1,\dots ,a_n). \end{aligned}$$

Then,

$$\begin{aligned} \begin{aligned} \min _{\begin{array}{c} k \in \Omega _h \\ i \in \Omega _v \end{array}} \frac{a_i b_k c_k V_i^* }{\gamma _k H_k} \cdot \mathscr {D}^{-1} \check{L}\mathscr {A}_2 \le \mathscr {D}^{-1} \mathscr {B} \mathscr {C}^{-1}\mathscr {A} \le \max _{\begin{array}{c} k \in \Omega _h \\ i\in \Omega _v \end{array}} \frac{a_ib_k c_k V_i^* }{\gamma _k H_k} \cdot \mathscr {D}^{-1}\check{L}\mathscr {A}_2. \end{aligned} \end{aligned}$$

A similarity transformation for $\mathscr {D}^{-1}\check{L}\mathscr {A}_2$ yields

$$\begin{aligned} \check{E}=(\check{e}_{ij})_{n \times n}=U \mathscr {D}^{-1} \check{L}\mathscr {A}_2 U^{-1}, \end{aligned}$$

where $U=\textrm{diag}(\mu _1, \dots , \mu _n)$. So, $\rho (\check{E})=\rho ( \mathscr {D}^{-1} \check{L}\mathscr {A}_2 )$ and

$$\begin{aligned} \begin{aligned} \min _{\begin{array}{c} k \in \Omega _h \\ i \in \Omega _v \end{array}} \sqrt{ \frac{a_i b_k c_k V_i^* }{\gamma _k H_k }} \cdot \sqrt{\rho ( \check{E} ) } \le \mathcal {R}_0 \le \max _{\begin{array}{c} k \in \Omega _h \\ i \in \Omega _v \end{array}} \sqrt{ \frac{a_i b_k c_k V_i^* }{\gamma _k H_k}} \cdot \sqrt{\rho ( \check{E} ) }. \end{aligned} \end{aligned}$$

Since

$$\begin{aligned} \check{l}_{ij}= \sum _{k \in \Omega _h} \left( \dfrac{ p_{ki}h_k }{h_i^* } \cdot \dfrac{ p_{kj} h_k }{h_j^* } \right) \le \sum _{k \in \Omega _h} \dfrac{ p_{ki}h_k }{h_i^* }=1,\quad \forall i,\, j\in \Omega _v, \end{aligned}$$

then

$$\begin{aligned} \begin{aligned} \varvec{1} \check{E}= \varvec{1} U \mathscr {D}^{-1} \check{L}\mathscr {A}_2 U^{-1} = \varvec{1} \check{L} \mathscr {A}_2 U^{-1}\le n\varvec{1}\mathscr {A}_2 U^{-1} \le \max _{j\in \Omega _v}\frac{a_j}{\mu _j}\cdot n\varvec{1}. \end{aligned} \end{aligned}$$

Therefore, it follows from Theorem 8.1.22 in Horn and Johnson (2013) that

$$\begin{aligned} \rho (\check{E}) \le \max _{j\in \Omega _v}\frac{a_j}{\mu _j}\cdot n, \end{aligned}$$

which implies

$$\begin{aligned} \mathcal {R}_0\le \max _{\begin{array}{c} k \in \Omega _h \\ i \in \Omega _v \end{array}} \sqrt{ \frac{a_i b_k c_k V_i^* }{\gamma _k H_k}} \cdot \max _{j\in \Omega _v}\sqrt{\frac{a_j}{\mu _j}}\cdot \sqrt{n} =\sqrt{n} \max _{\begin{array}{c} k \in \Omega _h \\ i,\,j \in \Omega _v \end{array}} \tilde{\mathcal {R}}_{ijk}. \end{aligned}$$

This completes the proof. $\square$

#### Remark 4.11

Inspired by the corresponding Lagrangian model (see Theorem 4.9 in Gao and Cao (2024)), if $\Omega _v^0=\Omega _v$, then the lower bound estimate

$$\begin{aligned} \mathcal {R}_0 \ge \sqrt{\dfrac{n}{m}}\min _{\begin{array}{c} k \in \Omega _h \\ i,\,j \in \Omega _v \end{array}} \tilde{\mathcal {R}}_{ijk}, \end{aligned}$$

may hold. Following the proof of Theorem 4.10, it suffices to show that $\varvec{1}\check{L}\ge \frac{m}{n}\varvec{1}$. Unfortunately, this is not always true. For example, consider a two-group two-patch environment with

$$\begin{aligned} h_1=\frac{3}{4},\quad h_2=\frac{1}{4},\quad p_{11}=\frac{1}{8},\quad p_{12}=\frac{7}{8}, \quad p_{21}=\frac{3}{4},\quad p_{22}=\frac{1}{4}, \end{aligned}$$

then

$$\begin{aligned} \check{L}=\begin{pmatrix} \frac{5}{9} &{} \frac{25}{69} \\ \frac{25}{69} &{} \frac{445}{529} \end{pmatrix}\ \text{ and } \ \varvec{1}\check{L}=\begin{pmatrix} \frac{190}{207}&\frac{1910}{1587} \end{pmatrix}\ngeq \varvec{1}, \end{aligned}$$

even though the inequalities $\rho (\check{L})\ge \frac{n}{m}$ and $\sum _{i,j\in \Omega _v}\check{l}_{ij}\ge \frac{n^2}{m}$ hold for *m*-group *n*-patch environment by the proof of Theorem 4.12 in Gao and Cao (2024).

In a homogeneous environment with *m* host groups and *n* vector patches, it follows from (3.4) that the basic reproduction number of patch $k\in \Omega _v$ in disconnection takes the form

$$\begin{aligned} \mathcal {R}_0^{(k)}(m/n)= & {} \sqrt{\frac{a^2 V_k^*}{\mu ( \sum _{l \in \Omega _h} p_{lk} H_l)^2 } \sum \limits _{i \in \Omega _h} \dfrac{b c p_{ik} H_i}{\gamma }}\\= & {} \sqrt{\dfrac{a^2bcV}{\mu \gamma H}} \cdot \sqrt{\dfrac{v_k^*}{h_k^*}} =\mathcal {R}_0(1/1)\cdot \sqrt{\dfrac{v_k^*}{h_k^*}}. \end{aligned}$$

Next, we use $\mathcal {R}_0^{(k)}(m/n)$ to estimate the basic reproduction number $\mathcal {R}_0(m/n)$.

#### Proposition 4.12

Let $\mathcal {R}_0(m/n)$ be the basic reproduction number of model (2.3) in a homogeneous environment with *m* host groups and *n* vector patches. If $\Omega _v^0=\Omega _v$, then

$$\begin{aligned} \min _{k \in \Omega _v} \mathcal {R}_0^{(k)}(m/n) \le \mathcal {R}_0(m/n) \le \max _{k \in \Omega _v} \mathcal {R}_0^{(k)}(m/n). \end{aligned}$$

#### Proof

In a homogeneous environment, according to the proof of Corollary 4.4, we have

$$\begin{aligned} \mathcal {R}_0(m/n)=\sqrt{\frac{a^2 b c V }{\gamma H}}\cdot \sqrt{ \rho ( \hat{L}) }, \end{aligned}$$

where $$\hat{L}=\mathscr {D}^{-1}\mathscr {B}_1 \mathscr {B}_2 \mathscr {A}_1$$ with

$$\begin{aligned} \mathscr {D}=\mu {\mathbb {I}}-D,\quad \hat{\mathscr {B}}=\mathscr {B}_1\mathscr {B}_2 = \left( \frac{ p_{ki} v_i^* }{h_i^*} \right) _{n \times m},\quad \mathscr {A}_1=\left( \frac{ p_{kj} h_k }{h_j^*}\right) _{m \times n}. \end{aligned}$$

It follows from

$$\begin{aligned} \hat{\mathscr {B}}=\textrm{diag}\left\{ \frac{v_1^* }{h_1^*},\dots ,\frac{v_n^* }{h_n^*}\right\} P^{\textrm{T}}, \end{aligned}$$

that

$$\begin{aligned} \min _{k \in \Omega _v}\frac{v_k^*}{h_k^*}\cdot \mathscr {D}^{-1}P^{\textrm{T}} \mathscr {A}_1\le \hat{L} \le \max _{k \in \Omega _v}\frac{v_k^*}{h_k^*} \cdot \mathscr {D}^{-1}P^{\textrm{T}} \mathscr {A}_1. \end{aligned}$$

The fact $\varvec{1}\mathscr {D}^{-1}P^{\textrm{T}}\mathscr {A}_1=\frac{1}{\mu }\varvec{1}$ implies $\rho (\mathscr {D}^{-1}P^{\textrm{T}}\mathscr {A}_1)=\frac{1}{\mu }$ and hence

$$\begin{aligned} \min _{k \in \Omega _v}\frac{v_k^*}{h_k^*}\cdot \frac{1}{\mu }\le \rho (\hat{L}) \le \max _{k \in \Omega _v}\frac{v_k^*}{h_k^*}\cdot \frac{1}{\mu }, \end{aligned}$$

A combination of expressions of $\mathcal {R}_0^{(k)}(m/n)$ and $\mathcal {R}_0(m/n)$ completes the proof. $\square$

Comparing the three estimates of $\mathcal {R}_0(m/n)$ with respect to $\mathcal {R}_{ijkr}, \tilde{\mathcal {R}}_{ijk}$ and $\mathcal {R}_0^{(k)}$, we can see that, in a homogenous environment, the lower bound given by Corollary 4.4 is the greatest while its upper bound is sharper than that of Theorem 4.10 but may or may not be better than that of Proposition 4.12. When only vectors or hosts move, one can refer to Gao and Ruan (2011), Gao et al. (2019) and Gao and Cao (2024) for some additional estimates.

### Minimization of $\mathcal {R}_0$ via vector control

In this subsection, we will focus on reducing disease persistence through allocating limited resource for vector control. Specifically, if a fixed number of vectors can be culled, then how many vectors are eliminated by each patch to minimize the reproduction number. Due to the complexity of this optimization problem, we just consider a two-group two-patch homogeneous environment with only hosts move between patches ($D={\mathbb {I}}_2$ and $\Omega _v^0=\Omega _v$). In this case, the model (2.3) can be rewritten as

4.5 $$\begin{aligned} \begin{aligned} \frac{dI_i^h}{dt}&=b \sum _{k=1}^2 a \frac{I_k^v}{p_{1k}H_1+p_{2k}H_2} p_{ik} (H_i - I_i^h ) - \gamma I_i^h,\quad i \in \Omega _h=\{1,2\}, \\ \frac{dI_j^v}{dt}&=a c\frac{ p_{1j}I_1^h+p_{2j}I_2^h}{p_{1j}H_1+p_{2j}H_2} (\tilde{V_j} - I_j^v ) - \mu I_j^v,\quad j \in \Omega _v=\{1,2\}, \\ \end{aligned} \end{aligned}$$

where $\tilde{V}_j\in [0,V_j]$ represents the vector population size of patch *j* after vector control. Using the next generation matrix method (Diekmann et al. 1990; van den Driessche and Watmough 2002), the control reproduction number of model (4.5) is

$$\begin{aligned} \begin{aligned} \mathcal {R}_c=\sqrt{\dfrac{a^2bc\tilde{V}}{\mu \gamma H}} \cdot \sqrt{\rho (\tilde{\mathscr {B}}\tilde{\mathscr {A}})} =\tilde{\mathcal {R}}_0(1/1)\cdot \sqrt{\rho (\tilde{\mathscr {B}}\tilde{\mathscr {A}})}, \end{aligned} \end{aligned}$$

where

$$\begin{aligned} \begin{aligned} \tilde{\mathscr {B}}&=\left( \tilde{b}_{ik} \right) _{2 \times 2}=\left( \dfrac{p_{ki} \tilde{v}_i}{h_i^*} \right) _{2 \times 2}\quad \text{ and } \quad \tilde{\mathscr {A}}&=\left( \tilde{a}_{kj} \right) _{2 \times 2}=\left( \dfrac{p_{kj}h_k}{h_j^*} \right) _{2 \times 2}, \end{aligned} \end{aligned}$$

and

$$\begin{aligned} \tilde{V}=\tilde{V}_1+\tilde{V}_2\quad \text{ and }\quad \tilde{v}_i=\frac{\tilde{V}_i}{\tilde{V}},\ \ i\in \{1,2\}. \end{aligned}$$

Since

$$\begin{aligned} \begin{aligned} \tilde{\mathscr {B}} = \begin{pmatrix} \dfrac{p_{11}\tilde{v}_1}{h_1^*} &{} \dfrac{p_{21}\tilde{v}_1}{h_1^*} \\ \dfrac{p_{12}\tilde{v}_2}{h_2^*} &{} \dfrac{p_{22}\tilde{v}_2}{h_2^*} \end{pmatrix} =\begin{pmatrix} \tilde{v}_1 &{} \\ &{} \tilde{v}_2 \end{pmatrix} \begin{pmatrix} \dfrac{1}{h_1^*} &{} \\ &{} \dfrac{1}{h_2^*} \end{pmatrix} \begin{pmatrix} p_{11} &{} p_{21} \\ p_{12} &{} p_{22} \end{pmatrix} \end{aligned} \end{aligned}$$

and

$$\begin{aligned} \begin{aligned} \tilde{\mathscr {A}} =\begin{pmatrix} \dfrac{p_{11}h_1}{h_1^*} &{} \dfrac{p_{12}h_1}{h_2^*} \\ \dfrac{p_{21}h_2}{h_1^*} &{} \dfrac{p_{22}h_2}{h_2^*} \end{pmatrix} =\begin{pmatrix} h_1 &{} \\ &{} h_2 \end{pmatrix} \begin{pmatrix} p_{11} &{} p_{12} \\ p_{21} &{} p_{22} \end{pmatrix} \begin{pmatrix} \dfrac{1}{h_1^*} &{} \\ &{} \dfrac{1}{h_2^*} \end{pmatrix}, \end{aligned} \end{aligned}$$

we have

$$\begin{aligned} \begin{aligned} \tilde{\mathscr {B}} \tilde{\mathscr {A}} =\begin{pmatrix} \tilde{v}_1 &{} \\ &{} \tilde{v}_2 \end{pmatrix} \begin{pmatrix} \dfrac{1}{h_1^*} &{} \\ &{} \dfrac{1}{h_2^*} \end{pmatrix} P^{\textrm{T}} \begin{pmatrix} h_1 &{} \\ &{} h_2 \end{pmatrix} P \begin{pmatrix} \dfrac{1}{h_1^*} &{} \\ &{} \dfrac{1}{h_2^*} \end{pmatrix}. \end{aligned} \end{aligned}$$

Minimizing $\mathcal {R}_c$ is equivalent to minimizing $\rho (\tilde{\mathscr {B}}\tilde{\mathscr {A}})$. Note that

$$\begin{aligned} \begin{aligned} \rho ( \tilde{\mathscr {B}} \tilde{\mathscr {A}} ) =\rho (D_v M ), \end{aligned} \end{aligned}$$

where

$$\begin{aligned} \begin{aligned} D_v =\begin{pmatrix} \tilde{v}_1 &{} \\ &{} \tilde{v}_2 \end{pmatrix} \end{aligned} \end{aligned}$$

and

$$\begin{aligned} \begin{aligned} M&=\begin{pmatrix} m_{11} &{} m_{12} \\ m_{21} &{} m_{22} \end{pmatrix}:=\begin{pmatrix} \dfrac{1}{(h_1^*)^2} &{} \\ &{} \dfrac{1}{(h_2^*)^2} \end{pmatrix} P^\mathrm{{T}} \begin{pmatrix} h_1 &{} \\ &{} h_2 \end{pmatrix} P\\&= \begin{pmatrix} \dfrac{p_{11}^2 h_1 + p_{21}^2 h_2}{(h_1^*)^2} &{} \dfrac{p_{11}p_{12} h_1 + p_{21}p_{22} h_2}{(h_1^*)^2} \\ \dfrac{p_{11}p_{12} h_1 + p_{21}p_{22} h_2}{(h_2^*)^2} &{} \dfrac{p_{12}^2 h_1 + p_{22}^2 h_2}{(h_2^*)^2} \end{pmatrix}. \end{aligned} \end{aligned}$$

Assume that the host residence times matrix *P* satisfies

($$\mathcal {H}2$$)
$$\det (P)=p_{11}+p_{22}-1\ne 0$$, $$P\ne {\mathbb {I}}_2$$ and $$P\ne \begin{pmatrix} 0 &{} 1\\ 1 &{} 0 \end{pmatrix}$$.

Otherwise, $p_{11}=p_{21}$ and $p_{12}=p_{22}$, i.e., the two host groups spend the same proportion of time on the same patch, or $p_{11}=1$ and $p_{21}=0$, or $p_{11}=0$ and $p_{21}=1$, which implies that

$$\begin{aligned} \begin{aligned} \rho ( \tilde{\mathscr {B}} \tilde{\mathscr {A}} ) \equiv \left\{ \begin{array}{ll} 1,\quad &{}\text{ if } \ 0<p_{11}=p_{21}<1, \\ \tilde{v}_1,\quad &{}\text{ if } \ p_{11}=p_{21}=1, \text{ i.e., } \Omega _v^0=\{1\}, \\ \tilde{v}_2,\quad &{}\text{ if } \ p_{11}=p_{21}=0, \text{ i.e., } \Omega _v^0=\{2\}, \\ \max \{\frac{\tilde{v}_1}{h_1},\frac{\tilde{v}_2}{h_2}\},\quad &{}\text{ if } \ p_{11}=1\ \text{ and } \ p_{21}=0, \\ \max \{\frac{\tilde{v}_1}{h_2},\frac{\tilde{v}_2}{h_1}\},\quad &{}\text{ if } \ p_{11}=0\ \text{ and } \ p_{21}=1, \end{array}\right. \end{aligned} \end{aligned}$$

holds for vector control strategy $(\tilde{v}_1,\tilde{v}_2)$ by Remarks 4.8 and 4.9 or a direct calculation of $\rho (D_vM)$. In fact, the assumption $(\mathcal {H}2)$ guarantees that the host-vector network associated to model (4.5) is strongly connected (Gao and Cao 2024). We first get some properties of the matrix *M* which are independent of $(\tilde{v}_1,\tilde{v}_2)$.

#### Lemma 4.13

Suppose $(\mathcal {H}2)$ is valid for model (4.5). The matrix *M* has the following properties:

1. $$m_{ij}>0$$, $$1 \le i,\,j \le 2$$;
2. $$m_{11}^2 > m_{12}m_{21}$$ and $$m_{22}^2 > m_{12}m_{21}$$;
3. $$\det (M)=m_{11}m_{22}-m_{12}m_{21}>0$$;
4. $$m_{11}+m_{12}=\dfrac{1}{h_1^*}$$ and $$m_{21}+m_{22}=\dfrac{1}{h_2^*}$$, and $$\dfrac{1}{m_{11}+m_{12}}+\dfrac{1}{m_{21}+m_{22}}=1$$;
5. $$\textrm{sgn}( m_{11}-m_{22} ) =\textrm{sgn}( m_{12}-m_{21} )=\textrm{sgn}(h_2^*-h_1^* ) $$;
6. $$\left( \dfrac{m_{11}+m_{12}}{m_{21}+m_{22}} \right) ^2 = \dfrac{m_{12}}{m_{21}}=\left( \dfrac{h_2^*}{h_1^*}\right) ^2 $$.

#### Proof

(C1) If $m_{11}=0$ or $m_{22}=0$, then $p_{11}=p_{21}=0$ or $p_{12}=p_{22}=0$, a contradiction to ($\mathcal {H}2$). If $m_{12}=0$ or $m_{21}=0$, then $p_{11}p_{12}=p_{21}p_{22}=0$ and hence

$$\begin{aligned} P\in \left\{ \begin{pmatrix} 1 &{} 0 \\ 0 &{} 1 \end{pmatrix},\ \begin{pmatrix} 0 &{} 1 \\ 0 &{} 1 \end{pmatrix},\ \begin{pmatrix} 1 &{} 0 \\ 1 &{} 0 \end{pmatrix},\ \begin{pmatrix} 0 &{} 1 \\ 1 &{} 0 \end{pmatrix}\right\} , \end{aligned}$$

which again contradicts to ($\mathcal {H}2$).

(C2) Since

$$\begin{aligned} \begin{aligned} m_{11}^2&=\left( \dfrac{p_{11}^2 h_1 + p_{21}^2 h_2}{(h_1^*)^2} \right) ^2> m_{12}m_{21}\\&=\left( \dfrac{p_{11}p_{12} h_1 + p_{21}p_{22} h_2}{h_1^*h_2^*}\right) ^2 \\&\Leftrightarrow \quad ( p_{11}^2 h_1 + p_{21}^2 h_2 )h_2^* > ( p_{11}p_{12} h_1 + p_{21}p_{22} h_2 ) h_1^*, \end{aligned} \end{aligned}$$

then by assumption ($\mathcal {H}2$) we have

$$\begin{aligned} \begin{aligned}&\qquad ( p_{11}^2 h_1 + p_{21}^2 h_2 )h_2^* - ( p_{11}p_{12} h_1 + p_{21}p_{22} h_2 )h_1^* \\&= ( p_{11}^2 h_1 + p_{21}^2 h_2 ) ( p_{12}h_1+p_{22}h_2 ) - ( p_{11}p_{12} h_1 + p_{21}p_{22} h_2 )( p_{11}h_1+p_{21}h_2 )\\&= ( ( p_{11}^2p_{22}+p_{21}^2p_{12})- ( p_{11}p_{12}p_{21}+p_{11}p_{21}p_{22} ) )h_1h_2\\&=(p_{11}-p_{21})(p_{11}p_{22}-p_{12}p_{21})h_1h_2\\&=(p_{11}+p_{22}-1)^2h_1h_2>0. \end{aligned} \end{aligned}$$

Similarly, the inequality $m_{22}^2 > m_{12}m_{21}$ can be proved.

(C3) The result follows from (C1) and (C2) or a direct calculation of

$$\begin{aligned} \begin{aligned} \det (M)&= \begin{vmatrix} \dfrac{1}{(h_1^*)^2}&\\&\dfrac{1}{(h_2^*)^2} \end{vmatrix} \cdot \det (P^\mathrm{{T}}) \cdot \begin{vmatrix} h_1&\\&h_2 \end{vmatrix} \cdot \det (P) =\dfrac{h_1 h_2}{(h_1^*h_2^*)^2} \left( \det (P) \right) ^2>0. \end{aligned} \end{aligned}$$

(C4) Direct calculation gives

$$\begin{aligned} \begin{aligned} M \varvec{1}^{\textrm{T}}&=\begin{pmatrix} \dfrac{1}{(h_1^*)^2} &{} \\ &{} \dfrac{1}{(h_2^*)^2} \end{pmatrix} P^\mathrm{{T}} \begin{pmatrix} h_1 &{} \\ &{} h_2 \end{pmatrix} P \begin{pmatrix} 1 \\ 1 \end{pmatrix}=\begin{pmatrix} \dfrac{1}{(h_1^*)^2} &{} \\ &{} \dfrac{1}{(h_2^*)^2} \end{pmatrix} P^\mathrm{{T}}\begin{pmatrix} h_1 \\ h_2 \end{pmatrix} \\&= \begin{pmatrix} \dfrac{1}{(h_1^*)^2} &{} \\ &{} \dfrac{1}{(h_2^*)^2} \end{pmatrix} \begin{pmatrix} h_1^* \\ h_2^* \end{pmatrix} =\begin{pmatrix} \dfrac{1}{h_1^*} \\ \dfrac{1}{h_2^*} \end{pmatrix}=\begin{pmatrix} m_{11}+m_{12} \\ m_{21}+m_{22} \end{pmatrix}. \end{aligned} \end{aligned}$$

(C5) A simple computation yields

$$\begin{aligned} \begin{aligned} m_{12}-m_{21}&=\dfrac{ p_{11}p_{12} h_1 + p_{21}p_{22} h_2 }{(h_1^*h_2^*)^2}(h_2^*-h_1^*). \end{aligned} \end{aligned}$$

It follows from (C4) that

$$\begin{aligned} \left( m_{11}+m_{12}\right) - \left( m_{21}+m_{22}\right) =\dfrac{1}{h_1^*}-\dfrac{1}{h_2^*}, \end{aligned}$$

which implies

$$\begin{aligned} \begin{aligned}&\qquad m_{11}-m_{22}=\left( \dfrac{1}{h_1^*}-\dfrac{1}{h_2^*} \right) -\left( m_{12}-m_{21} \right) \\&= \dfrac{h^*_2-h^*_1}{h^*_1 h^*_2}-\dfrac{ p_{11}p_{12} h_1 + p_{21}p_{22} h_2 }{(h_1^*h_2^*)^2}(h_2^*-h_1^*) \\&= \dfrac{h^*_2-h^*_1}{(h^*_1h^*_2)^2} \left( h^*_1h^*_2- \left( p_{11}p_{12} h_1 + p_{21}p_{22} h_2 \right) \right) \\&= \dfrac{h^*_2-h^*_1}{(h^*_1h^*_2)^2} \left( \left( p_{11}h_1+p_{21}h_2 \right) \left( p_{12}h_1+p_{22}h_2 \right) - \left( p_{11}p_{12} h_1 + p_{21}p_{22} h_2\right) (h_1+h_2) \right) \\&= \dfrac{h^*_2-h^*_1}{(h^*_1h^*_2)^2} \left( p_{11}p_{22}+p_{12}p_{21}-p_{11}p_{12} - p_{21}p_{22}\right) h_1h_2\\&= \dfrac{h^*_2-h^*_1}{(h^*_1h^*_2)^2}h_1h_2 (p_{11}-p_{21})(p_{22}-p_{12}) \\&= \dfrac{h^*_2-h^*_1}{(h^*_1h^*_2)^2}h_1h_2 (p_{11}+p_{22}-1)^2. \end{aligned} \end{aligned}$$

Therefore, $$\textrm{sgn}\left( m_{11}-m_{22} \right) =\textrm{sgn}\left( m_{12}-m_{21} \right) =\textrm{sgn}\left( h^*_2-h^*_1 \right) $$.

(C6) It can be obtained by using (C4) and the explicit expressions of $m_{12}$ and $m_{21}$. $\square$

The total number of vectors being culled over the two patches is

$$\begin{aligned} \eta :=V-\tilde{V}=(V_1+V_2)-(\tilde{V}_1+\tilde{V}_2) \in [0,\,V], \end{aligned}$$

where patches 1 and 2 cull $V_1-\tilde{V}_1$ and $V_2-\tilde{V}_2$ vectors, respectively. Thus, the number of vectors on patches 1 and 2 after control are

$$\begin{aligned} \tilde{V}_1 \quad \textrm{and} \quad \tilde{V}_2=V-\eta -\tilde{V}_1, \end{aligned}$$

respectively. The nonnegativity of $\tilde{V}_1$ and $\tilde{V}_2$ requires that

$$\begin{aligned} \max \{0,V_1-\eta \} \le \tilde{V}_1 \le \min \{V_1,V-\eta \}. \end{aligned}$$

Therefore, the proportion of vectors living in patch 1 after control, denoted by *x*, satisfies

$$\begin{aligned} x_0:=\max \left\{ 0,\frac{V_1-\eta }{V-\eta }\right\} \le x:=\tilde{v}_1=\frac{\tilde{V}_1}{\tilde{V}}=\frac{\tilde{V}_1}{V-\eta } \le x^0:=\min \left\{ \frac{V_1}{V-\eta },1\right\} \end{aligned}$$

and the matrix $D_v$ can be written as

$$\begin{aligned}D_v =\begin{pmatrix} \tilde{v}_1 &{} \\ &{} \tilde{v}_2 \end{pmatrix}= \begin{pmatrix} x &{} \\ &{} 1-x \end{pmatrix},\quad x\in [x_0,x^0]. \end{aligned}$$

For fixed $\eta \in [0,V]$, we will find *x* such that $\mathcal {R}_c(x)$ attains its minimum, i.e.,

$$\begin{aligned} \begin{aligned} \mathop {\mathrm {arg\,min}}\limits \limits _{x\in [x_0,x^0]} \mathcal {R}_{c}(x):=\left\{ x \in [x_0,x^0]:\ \mathcal {R}_c(s)\ge \mathcal {R}_c(x),\ \forall s\in [x_0,x^0] \right\} , \end{aligned} \end{aligned}$$

which corresponds to the culling of

$$\begin{aligned} X:=V_1-x \tilde{V}=V_1-x (V-\eta ) \end{aligned}$$

vectors in patch 1. It follows from Corollary 4.4 that $\mathcal {R}_c\ge \tilde{\mathcal {R}}_0(1/1)$, i.e., $\rho ( \tilde{\mathscr {B}} \tilde{\mathscr {A}} ) =\rho (D_v M )\ge 1$, always holds for a homogeneous environment with no host-free patch. In particular, if the distributions of hosts and vectors are consistent across patches after control, that is,

$$\begin{aligned} \begin{aligned} \dfrac{\tilde{v}_1}{h_1^*}=\frac{x}{\frac{1}{m_{11}+m_{12}}} =\dfrac{\tilde{v}_2}{h_2^*}=\frac{1-x}{\frac{1}{m_{21}+m_{22}}}=1,\ \text{ i.e., } \ x=h_1^*, \end{aligned} \end{aligned}$$

then $\mathcal {R}_c=\tilde{\mathcal {R}}_0(1/1)$. We find that this is also a necessary condition.

#### Lemma 4.14

Suppose $(\mathcal {H}2)$ is valid for model (4.5). The equality $\mathcal {R}_c'(x^*)=0$ holds for some $x^*\in (x_0,x^0)$ if and only if $x^*=h_1^*\in (x_0,x^0)$, i.e., any critical number of $\mathcal {R}_c(x)$ corresponds to consistent distribution of hosts and vectors and a global minimum.

#### Proof

Since only $D_v$ contains *x*, the relation

$$\begin{aligned} \begin{aligned} \mathcal {R}_c=\tilde{\mathcal {R}}_0(1/1)\cdot \sqrt{\rho (D_v M)} \end{aligned} \end{aligned}$$

indicates that it suffices to show the result for $\tilde{\mathcal {R}}_{c}:=\rho (D_v M)$. Direct calculations give

$$\begin{aligned} \begin{aligned} D_v M=\begin{pmatrix} x &{} \\ &{} 1-x \end{pmatrix} \begin{pmatrix} m_{11} &{} m_{12} \\ m_{21} &{} m_{22} \end{pmatrix}=\begin{pmatrix} m_{11}x &{} m_{12} x\\ m_{21}(1-x) &{} m_{22}(1-x) \end{pmatrix}, \end{aligned} \end{aligned}$$

and its associated characteristic equation

$$\begin{aligned} \lambda ^2- ( m_{11}x+m_{22}(1-x))\lambda + ( m_{11} m_{22} - m_{12} m_{21} )x(1-x)=0. \end{aligned}$$

Thus,

$$\begin{aligned} \begin{aligned} \tilde{\mathcal {R}}_{c}=\dfrac{1}{2}( \Phi + \sqrt{\Psi }), \end{aligned} \end{aligned}$$

where

$$\begin{aligned} \begin{aligned} \Phi&= m_{11}x+m_{22}(1-x), \\ \Psi&= (m_{11}x-m_{22}(1-x))^2+4m_{12} m_{21}x(1-x). \end{aligned} \end{aligned}$$

Differentiating $\tilde{\mathcal {R}}_{c}$ with respect to *x* yields

$$\begin{aligned} \begin{aligned} \dfrac{\partial \tilde{\mathcal {R}}_{c} }{\partial x} = \dfrac{1}{2} \left( \Phi ' + \dfrac{\Psi ' }{2 \sqrt{\Psi }} \right) , \end{aligned} \end{aligned}$$

where

$$\begin{aligned} \begin{aligned} \Phi '&= m_{11}-m_{22},\\ \Psi '&= 2(m_{11}+m_{22}) ( m_{11}x-m_{22}(1-x))+4m_{12}m_{21} (1-2x). \end{aligned} \end{aligned}$$

A straightforward but tedious calculation gives

4.6 $$\begin{aligned} \begin{aligned} 4 \Psi \cdot (\Phi ')^2 -(\Psi ')^2=16\det (M) ((m_{11}x-m_{22}(1-x))^2-m_{12}m_{21}(1-2x)^2). \end{aligned} \nonumber \\ \end{aligned}$$

In what follows, we show that $\frac{\partial \tilde{\mathcal {R}}_{c} }{\partial x} =0$ only if $x=h_1^*$ in three cases.

*Case 1* $\Phi ' =0$ and $\Psi '=0$. It follows from $\Phi ' =0$ that $m_{11}= m_{22}$. By Lemma 4.13(C5), we have $h_1^*=h_2^*=\frac{1}{2}$. Moreover, by Lemma 4.13(C2), the assumption

$$\begin{aligned} \begin{aligned} \Psi '&=4m_{11}^2 ( 2x-1)+4m_{12}m_{21} (1-2x)=4 \left( m_{11}^2-m_{12}m_{21} \right) (2x-1)=0 \end{aligned} \end{aligned}$$

implies $$x= \frac{1}{2}$$. Thus, $$x= h_1^*$$.

*Case 2* $\Phi ' >0$, $\Psi '<0$, and $4 \Psi \cdot (\Phi ')^2 =(\Psi ')^2$. It follows from $\Phi ' >0$ that $m_{11}> m_{22}$. Again by Lemma 4.13(C5), we have $h_1^*<h_2^*$ and $m_{12}>m_{21}$. By Lemma 4.13(C3) and (4.6), the equality $4 \Psi \cdot (\Phi ')^2-(\Psi ')^2=0$ can be reduced to

4.7 $$\begin{aligned} \begin{aligned} m_{11}x-m_{22}(1-x)=\pm \sqrt{m_{12}m_{21}}(1-2x), \end{aligned} \end{aligned}$$

from which we have

4.8 $$\begin{aligned} \begin{aligned} x=\dfrac{m_{22} \pm \sqrt{m_{12}m_{21}} }{m_{11}+m_{22} \pm 2\sqrt{m_{12}m_{21}} } \, \in \left( 0,\frac{1}{2}\right) . \end{aligned} \end{aligned}$$

Substituting (4.7) into $\Psi '$ gives

$$\begin{aligned} \begin{aligned} \Psi '&=\pm 2(m_{11}+m_{22}) \sqrt{m_{12}m_{21}}(1-2x) +4m_{12}m_{21}( 1-2x) \\&=2\sqrt{m_{12}m_{21}}(1-2x)(2\sqrt{m_{12}m_{21}}\pm (m_{11}+m_{22})). \end{aligned} \end{aligned}$$

It follows from Lemma 4.13(C2) and (4.8) that

$$\begin{aligned} \begin{aligned} x=\dfrac{m_{22}- \sqrt{m_{12}m_{21}} }{m_{11}+m_{22}- 2\sqrt{m_{12}m_{21}} }. \end{aligned} \end{aligned}$$

Otherwise, it contradicts to the assumption $\Psi '<0$. It remains to check $x=h_1^*$. Note that

$$\begin{aligned} \begin{aligned}&\frac{1}{x}=\dfrac{m_{11}+m_{22}- 2\sqrt{m_{12}m_{21}} }{m_{22}- \sqrt{m_{12}m_{21}} }=1+\dfrac{m_{11}- \sqrt{m_{12}m_{21}} }{m_{22}- \sqrt{m_{12}m_{21}} }, \\&\frac{1}{h_1^*}=\frac{h_1^*+h_2^*}{h_1^*}=1+\frac{h_2^*}{h_1^*}=1+\dfrac{\sqrt{m_{12}}}{\sqrt{m_{21}}}, \end{aligned} \end{aligned}$$

where the second equality is due to Lemma 4.13(C6). Thus, $x=h_1^*$ is equivalent to

$$\begin{aligned} \begin{aligned}&\qquad \dfrac{m_{11}- \sqrt{m_{12}m_{21}} }{m_{22}- \sqrt{m_{12}m_{21}} }=\dfrac{\sqrt{m_{12}}}{\sqrt{m_{21}}}\\&\Leftrightarrow \ m_{11}\sqrt{m_{21}}-m_{21}\sqrt{m_{12}}=m_{22}\sqrt{m_{12}}-m_{12}\sqrt{m_{21}}\\&\Leftrightarrow \ (m_{11}+m_{12})\sqrt{m_{21}}=(m_{21}+m_{22})\sqrt{m_{12}}. \end{aligned} \end{aligned}$$

The last equality follows from Lemma 4.13(C6).

*Case 3* $\Phi ' <0$, $\Psi '>0$, $4 \Psi \cdot (\Phi ')^2 =(\Psi ')^2$. It follows from $\Phi ' <0$ that $m_{11}< m_{22}$. By Lemma 4.13(C5), we have $h_1^*>h_2^*$ and $m_{12}<m_{21}$. Similar to Case 2, the equality $4 \Psi \cdot (\Phi ')^2 =(\Psi ')^2$ and the positivity of $\Psi '$ imply that

$$\begin{aligned} \begin{aligned} x=\dfrac{m_{22}- \sqrt{m_{12}m_{21}} }{m_{11}+m_{22}- 2\sqrt{m_{12}m_{21}} }\in \left( \frac{1}{2},1\right) . \end{aligned} \end{aligned}$$

We can verify that $x=h_1^*$ in the same way. $\square$

Without loss of generality, assume that the infection risk of patch 1 in disconnection is higher than or equal to that of patch 2 before vector control, that is, $\mathcal {R}_0^{(1)}\ge \mathcal {R}_0^{(2)}$, or equivalently, $\frac{V_1}{h_1^*}\ge \frac{V_2}{h_2^*}$. Under this circumstance, to make the distributions of hosts and vectors consistent after control, the minimum number of vectors that need to be culled, denoted by $\eta ^*$, satisfies

$$\begin{aligned} \dfrac{V_1-\eta ^*}{h_1^*}=\dfrac{V_2}{h_2^*},\ \text{ i.e., } \ \eta ^*=V_1- \dfrac{h_1^*}{h_2^*}V_2. \end{aligned}$$

#### Theorem 4.15

Suppose $(\mathcal {H}2)$ and $\frac{V_1}{h_1^*}\ge \frac{V_2}{h_2^*}$ are valid for model (4.5). The following statements on the optimal vector control strategy in terms of the control reproduction number $\mathcal {R}_c$ hold:

1. If $0< \eta <\eta ^*$, then $\mathop {\mathrm {arg\,min}}\limits \limits _{x\in [x_0,x^0]} \mathcal {R}_{c}(x)=x_0=\frac{V_1-\eta }{V-\eta }$, i.e., only vectors in patch 1 are culled;
2. If $\eta ^* \le \eta < V$, then $\mathop {\mathrm {arg\,min}}\limits \limits _{x\in [x_0,x^0]} \mathcal {R}_{c}(x)=h_1^*$, i.e., vectors in both patches are culled such that the distributions of hosts and vectors after control are consistent. More specifically, $V_1-h_1^*(V-\eta )$ and $V_2-h_2^*(V-\eta )$ vectors are culled in patches 1 and 2, respectively.

#### Proof

For the first part, the condition $0<\eta <\eta ^*$ is equivalent to $x_0>h_1^*$. In fact,

$$\begin{aligned} \begin{aligned} 0<\eta <\eta ^*\Leftrightarrow&\dfrac{V_1-\eta }{h_1^*}>\dfrac{V_2}{h_2^*}\Leftrightarrow \dfrac{V_1-\eta }{h_1^*}>\dfrac{V_1-\eta +V_2}{h_1^*+h_2^*} =\dfrac{V-\eta }{1}>\dfrac{V_2}{h_2^*}\\ \Leftrightarrow&\frac{V_1-\eta }{V-\eta }=x_0>h_1^*. \end{aligned} \end{aligned}$$

It follows from Lemma 4.14 that $\mathcal {R}_c(x)$ or $\tilde{\mathcal {R}}_c(x)$ has no critical number on $x\in [x_0,x^0]$ as $0<\eta \le \eta ^*$. Thus, $\mathcal {R}_c'(x)>0$ or $<0$ for all $x\in [x_0,x^0]$. Since $\mathcal {R}_c(x)$ attains its global minimum at the unique critical number $x=h_1^*$, the derivative $\mathcal {R}_c'(x)$ on $[x_0,x^0]$ must be positive, i.e., $\mathcal {R}_c(x)$ is strictly increasing in $x\in [x_0,x^0]$ as $0<\eta \le \eta ^*$. The second part is easy to see from Lemma 4.14. $\square$

#### Remark 4.16

In other words, the optimal strategy is to make the vector-to-host ratio of the high-risk patch closest to that of the whole environment, namely

$$\begin{aligned} \mathop {\mathrm {arg\,min}}\limits \limits _{x\in [x_0,x^0]} \mathcal {R}_{c}(x)=\mathop {\mathrm {arg\,min}}\limits \limits _{x\in [x_0,x^0]} \left| \frac{x}{h_1^*}-\frac{1}{1}\right| =\mathop {\mathrm {arg\,min}}\limits \limits _{x\in [x_0,x^0]} |x-h_1^*|. \end{aligned}$$

Since $|(1-x)-h_2^*|=|(1-h_2^*)-x|=|x-h_1^*|$, the optimal strategy makes the vector-to-host ratios of patches 1 and 2 simultaneously closest to that of the whole environment. When $(\mathcal {H}2)$ fails, the optimal strategy can be easily derived from the explicit expression of $\rho ( \tilde{\mathscr {B}} \tilde{\mathscr {A}} )$.

## Numerical simulations

In this section, we will use numerical approach to further explore the role of movement on the spread of vector-borne diseases. More specifically, the dependence of the basic reproduction number and the total number of infected hosts on the host residence times matrix and the optimal vector control in homogeneous and heterogeneous environments will be analyzed.

For simplicity, we consider an environment with two host groups and two vector patches and no host-free patch, i.e., $\Omega _h=\Omega _v=\Omega _v^0=\{1, \, 2 \}$. The model (2.3) can be written as

5.1 $$\begin{aligned} \begin{aligned} \frac{dI_1^h}{dt}&=b_1 \left( \dfrac{a_1 I_1^v p_{11} }{p_{11}H_1+p_{21}H_2} + \dfrac{a_2 I_2^v p_{12} }{p_{12}H_1+p_{22}H_2} \right) \left( H_1 - I_1^h \right) - \gamma _1 I_1^h, \\ \frac{dI_2^h}{dt}&=b_2 \left( \dfrac{a_1 I_1^v p_{21} }{p_{11}H_1+p_{21}H_2} + \dfrac{a_2 I_2^v p_{22} }{p_{12}H_1+p_{22}H_2} \right) \left( H_2 - I_2^h \right) - \gamma _2 I_2^h, \\ \frac{dI_1^v}{dt}&=a_1 \frac{c_1 p_{11} I_1^h+c_2 p_{21} I_2^h}{p_{11}H_1+p_{21}H_2} \left( V_1^* - I_1^v \right) - \mu _1 I_1^v -d_{21}I_1^v+d_{12}I_2^v, \\ \frac{dI_2^v}{dt}&=a_2 \frac{c_1 p_{12} I_1^h+c_2 p_{22} I_2^h}{p_{12}H_1+p_{22}H_2} \left( V_2^* - I_2^v \right) - \mu _2 I_2^v +d_{21}I_1^v-d_{12}I_2^v, \\ \end{aligned} \end{aligned}$$

where

$$\begin{aligned} V_1^*=\dfrac{d_{12}}{d_{12}+d_{21}}V \quad \text{ and } \quad V_2^*=\dfrac{d_{21}}{d_{12}+d_{21}}V. \end{aligned}$$

The basic reproduction number of model (5.1) is

5.2 $$\begin{aligned} \begin{aligned} \mathcal {R}_0=\sqrt{\rho \left( \mathscr {A}\mathscr {D}^{-1} \mathscr {B}\mathscr {C}^{-1} \right) }, \end{aligned} \end{aligned}$$

where

$$\begin{aligned}&\mathscr {A}=\begin{pmatrix} \dfrac{a_1 b_1 p_{11}H_1 }{p_{11}H_1+p_{21}H_2} &{} \dfrac{a_2 b_1 p_{12}H_1 }{p_{12}H_1+p_{22}H_2} \\ \dfrac{a_1 b_2 p_{21}H_2 }{p_{11}H_1+p_{21}H_2} &{} \dfrac{a_2 b_2 p_{22}H_2 }{p_{12}H_1+p_{22}H_2} \end{pmatrix},\qquad{} & {} \mathscr {C}=\begin{pmatrix} \gamma _1 &{} 0\\ 0 &{} \gamma _2 \end{pmatrix}, \\&\mathscr {B}=\begin{pmatrix} \dfrac{a_1 c_1 p_{11}V_1^* }{p_{11}H_1+p_{21}H_2} &{} \dfrac{a_1 c_2 p_{21}V_1^* }{p_{11}H_1+p_{21}H_2} \\ \dfrac{a_2 c_1 p_{12}V_2^* }{p_{12}H_1+p_{22}H_2} &{} \dfrac{a_2 c_2 p_{22}V_2^* }{p_{12}H_1+p_{22}H_2} \end{pmatrix}, \qquad{} & {} \mathscr {D}=\begin{pmatrix} \mu _1+d_{21} &{} -d_{12}\\ -d_{21} &{} \mu _2+d_{12} \end{pmatrix}. \end{aligned}$$

In what follows, the selection of parameter values is mainly based on the malaria epidemiology (Craig et al. 1999; Ruan et al. 2008), and the default time unit is per day.

### Example 5.1

($\mathcal {R}_0$ vs *P*). Consider a homogeneous environment with the following parameter setting:

$$\begin{aligned}&a=0.3,\quad{} & {} b=0.4,\quad{} & {} c=0.3,\quad{} & {} \gamma =0.03,\quad{} & {} \mu =\frac{1}{14}, \\&d_{12}=0.1,\quad{} & {} d_{21}=0.2,\quad{} & {} H_1=20000,\quad{} & {} H_2=22000, \quad{} & {} V=10000. \end{aligned}$$

**Fig. 2:**
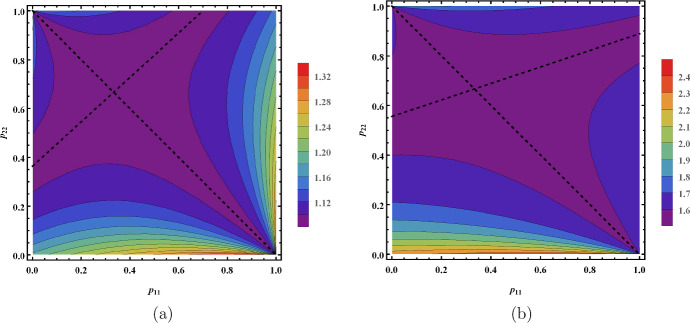
Contour plots of the basic reproduction number $\mathcal {R}_{0}$ of model (5.1) in terms of the residence time proportions $p_{11}$ and $p_{22}$ under two homogeneous environments. The black dashed lines are $L_1$ and $L_2$ on which $\mathcal {R}_0(p_{11}, p_{22})=\mathcal {R}_0(1/1)$

Then the vector population size of patches 1 and 2 are $V_1^*=3333$ and $V_2^*=6667$, respectively. Using (5.2), the contour plot of the basic reproduction number $\mathcal {R}_0$ with respect to $p_{11}$ and $p_{22}$, the proportions of time that hosts of groups 1 and 2 spend in patches 1 and 2, respectively, is plotted in Fig. 2a. The dependence of $\mathcal {R}_0$ on $p_{11}$ and $p_{22}$ is complicated. For example, for $p_{11}=0.2$, the dependence of $\mathcal {R}_0$ in $p_{22}$ is initially decreasing, then increasing, then decreasing, and finally increasing; for $p_{11}=0.8$, $\mathcal {R}_0$ initially decreases, then increases, and finally decreases in $p_{22}$. Using the same parameter set except that

$$\begin{aligned} H_1=5000 \quad \textrm{and} \quad H_2=15000, \end{aligned}$$

we similarly get the contour plot of $\mathcal {R}_0$ versus $p_{11}$ and $p_{22}$ as shown in Fig. 2b. Similarly, the dependence of $\mathcal {R}_0$ on $p_{11}$ and $p_{22}$ remains complicated.

Interestingly, in both panels of Fig. 2, the minimum value of $\mathcal {R}_0$, i.e., $\mathcal {R}_0(1/1)$, is taken at two straight lines, which form a kite-shaped area. Indeed, by Corollary 4.4, the one with positive slope is due to homogeneous mixing of hosts and vectors, i.e.,

$$\begin{aligned} \dfrac{V_1^*}{p_{11}H_{1}+p_{21}H_{2}}=\dfrac{V_1^*+V_2^*}{H_{1}+H_{2}} \ \Longleftrightarrow \ \dfrac{p_{11}H_{1}+(1-p_{22})H_{2}}{H_{1}+H_{2}}=\dfrac{d_{12}}{d_{12}+d_{21}}, \end{aligned}$$

which can be rewritten as

$$\begin{aligned} L_1: \, p_{22}=\dfrac{H_1}{H_2}p_{11}+1- \dfrac{d_{12}}{d_{12}+d_{21}} \left( 1+ \dfrac{H_1}{H_2} \right) . \end{aligned}$$

By Remark 4.8, if different host groups spend the same proportion of time on the same patch, i.e., $p_{11}=p_{21}$ and $p_{12}=p_{22}$, then $\mathcal {R}_0(2/2)=\mathcal {R}_0(1/1)$. So, the other line is

$$\begin{aligned} L_2: \, p_{22}=1-p_{11}. \end{aligned}$$

The presence of $L_1$ and $L_2$ means that $\mathcal {R}_0$ is always nonmonotone in terms of $p_{11}$ and $p_{22}$.

**Fig. 3:**
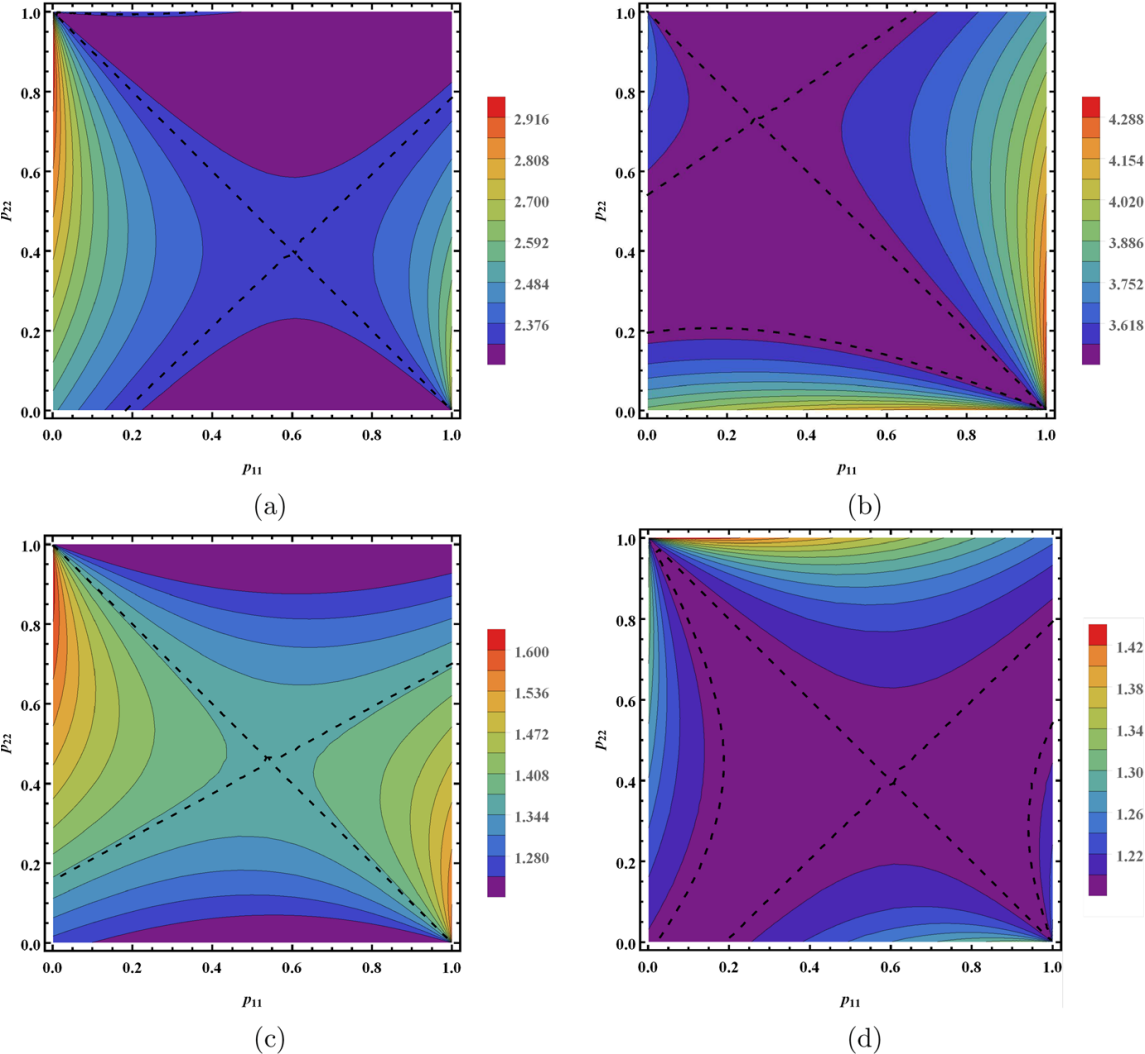
Contour plots of the basic reproduction number $\mathcal {R}_{0}$ of model (5.1) in terms of the residence time proportions $p_{11}$ and $p_{22}$ under four heterogeneous environments. The black dashed curves represent $\mathcal {R}_0(p_{11},p_{22})=\zeta =\mathcal {R}_0(p_{11},1-p_{11})$

When environment is heterogeneous, we select four parameter sets as listed in Table 1 and plot their corresponding contour plots of $\mathcal {R}_0$ versus $p_{11}$ and $p_{22}$ in Fig. 3. Note that

$$\begin{aligned} \mathcal {R}_0\equiv \zeta :=\sqrt{\frac{(b_1c_1\gamma _2H_1+b_2c_2\gamma _1H_2) (a_1^2\mu _2d_{12}+a_2^2\mu _1d_{21}+(a_1d_{12}+a_2d_{21})^2)V}{\gamma _1\gamma _2(\mu _1d_{12}+\mu _2(\mu _1+d_{21}))(d_{12}+d_{21})(H_1+H_2)^2}} \end{aligned}$$

along the line $L_2$ because of

$$\begin{aligned} \mathscr {A}=\begin{pmatrix} \dfrac{a_1 b_1 H_1 }{H_1+H_2} &{} \dfrac{a_2 b_1 H_1 }{H_1+H_2} \\ \dfrac{a_1 b_2 H_2 }{H_1+H_2} &{} \dfrac{a_2 b_2 H_2 }{H_1+H_2} \end{pmatrix} \ \ \text{ and } \ \ \mathscr {B}=\begin{pmatrix} \dfrac{a_1 c_1 V_1^* }{H_1+H_2} &{} \dfrac{a_1 c_2 V_1^* }{H_1+H_2} \\ \dfrac{a_2 c_1 V_2^* }{H_1+H_2} &{} \dfrac{a_2 c_2 V_2^* }{H_1+H_2} \end{pmatrix}. \end{aligned}$$

The dashed curves represent the position where $\mathcal {R}_0(p_{11},p_{22})=\zeta$. We see that $\zeta$ is generally not the minimum value of $\mathcal {R}_0$ and the changing pattern of $\mathcal {R}_0$ is similar or more complicated than that in a homogeneous environment.

**Table 1:** The parameter settings of Fig. 3

	$a_1$	$a_2$	$b_1$	$b_2$	$c_1$	$c_2$	$\gamma _1$	$\gamma _2$	$\mu _1$	$\mu _2$	$d_{12}$	$d_{21}$	$H_1$	$H_2$	*V*
a	0.4	0.5	0.4	0.35	0.15	0.2	0.03	0.02	$\frac{1}{14}$	$\frac{1}{16}$	0.2	0.1	2	2.2	3
b	0.4	0.5	0.3	0.35	0.25	0.2	0.03	0.02	$\frac{1}{14}$	$\frac{1}{16}$	0.1	0.2	1	1.5	3
c	0.2	0.3	0.1	0.3	0.3	0.2	0.03	0.02	$\frac{1}{14}$	$\frac{1}{16}$	0.2	0.1	1	2	3
d	0.2	0.25	0.3	0.25	0.3	0.2	0.03	0.02	$\frac{1}{14}$	$\frac{1}{16}$	0.2	0.1	2	2	3

### Example 5.2

**Fig. 4:**
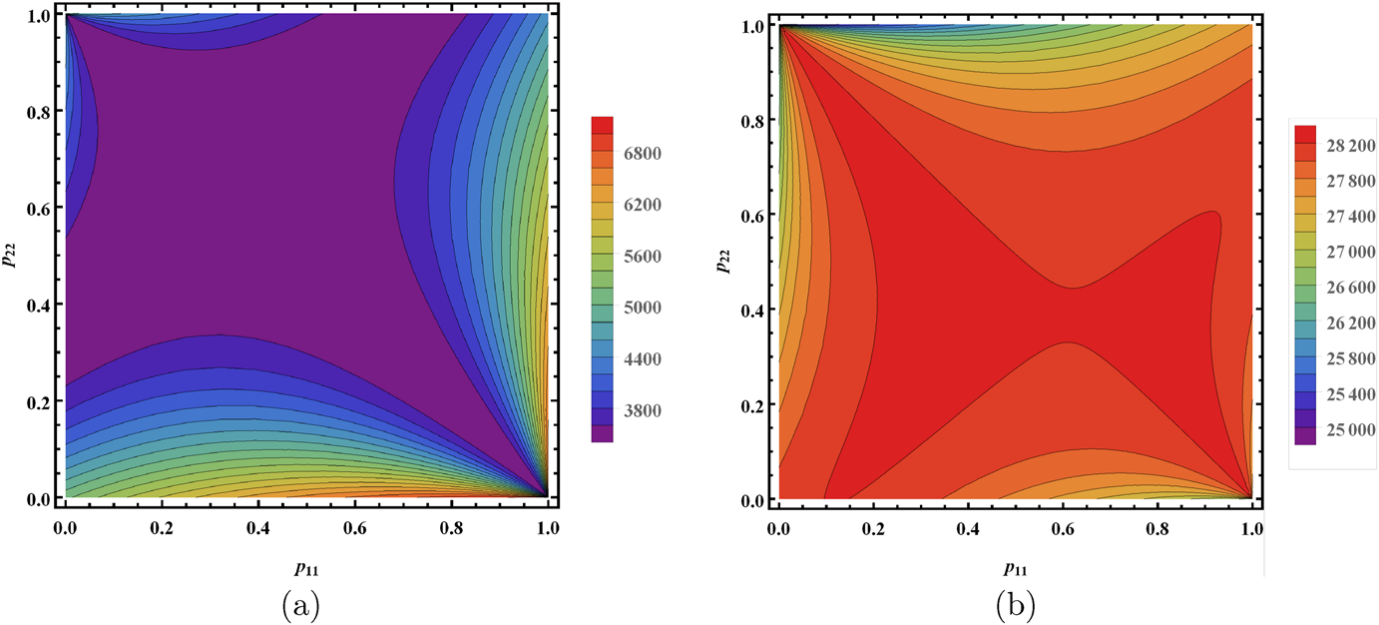
Contour plots of the total number of infected hosts ${I_1^h}^*+{I_2^h}^*$ versus the residence time proportions $p_{11}$ and $p_{22}$ in **a** homogeneous and **b** heterogeneous environments

(Total number of infected hosts versus *P*) The basic reproduction number $\mathcal {R}_0$ can be used to measure disease persistence. The larger the $\mathcal {R}_0$, the harder it is to eradicate the disease. However, there may exist inconsistency between disease persistence and disease prevalence (proportion of hosts being infected) in response to change in population movement (Gao and Lou 2021). Thus, it is necessary to explore the relationship between the total infected population and the proportions of residence time. Using the homogeneous parameter set in Fig. 2a and the heterogeneous parameter set in Fig. 3a, we illustrate the contour plot of the total host infection size $T_2:=I_1^{h*}+I_2^{h*}$ versus $p_{11}$ and $p_{22}$ at the unique endemic equilibrium $E^*$ in Fig. 4. The infection terms in model (5.1) are unaffected by *P* along the line $L_2$ and hence $T_2$ remains constant even in heterogeneous environment. Furthermore, in a homogeneous environment, along the lines $L_1$ and $L_2$ the total infection sizes of hosts and vectors $T_2=I^{h*}=I_1^{h*}+I_2^{h*}$ and $I^{v*}=I_1^{v*}+I_2^{v*}$ are constant, i.e.,

$$\begin{aligned} (I^{h*},I^{v*})=\left( \frac{a^2bcV-\gamma \mu H}{a^2bcV+ac\gamma H}H,\frac{a^2bcV-\gamma \mu H}{a^2bc+ab\mu }\right) , \end{aligned}$$

which is the positive equilibrium of the classical Ross–Macdonald model

$$\begin{aligned} \begin{aligned} \frac{dI^h}{dt}&=ab \frac{I^v}{H} (H - I^h ) - \gamma I^h, \\ \frac{dI^v}{dt}&=a c\frac{ I^h}{H} (V - I^v ) - \mu I^v. \end{aligned} \end{aligned}$$

Indeed, it is easy to verify that the endemic equilibrium is

$$\begin{aligned} E^*=(I_1^{h*},I_2^{h*},I_1^{v*},I_2^{v*})=\left( \frac{H_1}{H}I^{h*},\frac{H_2}{H}I^{h*}, \frac{V_1^*}{V}I^{v*},\frac{V_2^*}{V}I^{v*}\right) . \end{aligned}$$

Comparing Figs. 2a and 4a, we find that in a homogeneous environment, the change of $T_2$ in $p_{11}$ and $p_{22}$ is roughly consistent with that of $\mathcal {R}_0$. Moreover, both of them are small in the kite-shaped region, and large near $p_{11}=1$ and $p_{22}=0$. Surprisingly, when the total vector population size *V* increases to 30000, the reproduction number $\mathcal {R}_0$ is still small in the kite-shaped region but the total host infection size $T_2$ is large. By comparing Figs. 3a and 4b in a heterogeneous environment, it can be seen that $\mathcal {R}_0$ is relatively small but $T_2$ is very large in the kite-shaped region. These indicate that the inconsistency between disease persistence and host prevalence also occurs in the spatial spread of vector-borne diseases. Lowering the reproduction number should not be the only concern when disease elimination is impossible.

### Example 5.3

(Optimal vector control on $\mathcal {R}_0$) We consider how to minimize the control reproduction number $\mathcal {R}_c$ of model (4.5) through suitable allocation of limited vector control resources. Choosing a set of parameter values from a homogeneous environment:

$$\begin{aligned}&a=0.23,\quad b=0.4,\quad c=0.2,\quad \gamma =0.03,\quad \mu =\frac{1}{14},\quad d_{12}=d_{21}=0,\\&p_{11}=0.6,\quad p_{22}=0.8,\quad H_1=3000,\quad H_2=7000, \quad V_1=6000, \quad V_2=4000, \end{aligned}$$

then the effective host population sizes of patches 1 and 2 are respectively

$$\begin{aligned} p_{11}H_1+p_{21}H_2=3200 \quad \textrm{and } \quad p_{12}H_1+p_{22}H_2=6800. \end{aligned}$$

Therefore,

$$\begin{aligned} \frac{V_1}{p_{11}H_1+p_{21}H_2}=\frac{6000}{3200} >\frac{V_2}{p_{12}H_1+p_{22}H_2}=\frac{4000}{6800}, \end{aligned}$$

which means patch 1 has a higher risk of infection than patch 2. The basic reproduction number is $\mathcal {R}_0\approx 1.45$, so the disease persists in both patches.

**Fig. 5:**
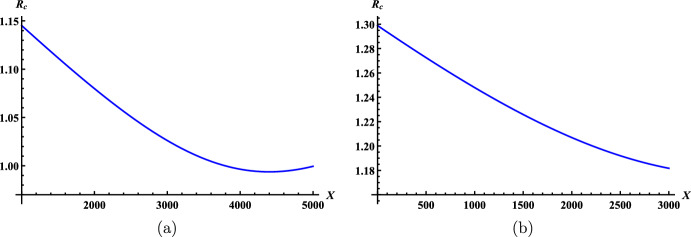
The control reproduction number $\mathcal {R}_c$ of model (4.5) versus the number of vectors being culled from the first patch, *X*, when the total number of vectors being culled is **a** $\eta =5000$, **b** $\eta =3000$

Now let us introduce vector control. The minimum number of vectors that need to be culled so that the distributions of hosts and vectors are consistent is $\eta ^*\approx 4118$. Suppose that $\eta =5000>\eta ^*$ vectors are to be culled from the two patches. The control reproduction number $\mathcal {R}_c$ in terms of the number of vectors being culled in patch 1, $X\in [1000,5000]$, is depicted in Fig. 5a. Clearly, $\mathcal {R}_c$ is initially decreasing then increasing in *X* and attains its minimum at $X=4400$. In fact, $X=4400$ means that the number of vectors remaining in patches 1 and 2 is 1600 and 3400, respectively, and the ratios of hosts to vectors in patches 1 and 2 are the same, i.e.,

$$\begin{aligned} \frac{V_1-X}{p_{11}H_1+p_{21}H_2}=\frac{1600}{3200} =\frac{V_2-(\eta -X)}{p_{12}H_1+p_{22}H_2}=\frac{3400}{6800}=\frac{1}{2}. \end{aligned}$$

**Fig. 6:**
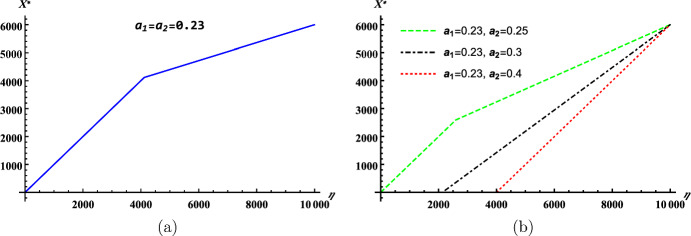
The optimal number of vectors being culled in patch 1, $X^*$, versus the total number of vectors being culled, $\eta$, in **a** homogeneous and **b** heterogeneous environment

It corresponds to minimum $\mathcal {R}_c\approx 0.99<1$, which means that an appropriate control strategy can achieve disease elimination. In case we choose $\eta =3000<\eta ^*$, then it is impossible to distribute vectors and hosts consistently. As shown in Fig. 5b, $\mathcal {R}_c$ strictly decreases with respect to *X* and reaches its minimum at $X=\eta$, i.e., the best strategy is to only eliminate vectors in patch 1. Furthermore, in Fig. 6a we plot $X^*$, the number of vectors being culled in patch 1 that minimizes $\mathcal {R}_c$, versus $\eta$, the total number of vectors culled over both patches. We can see that $X^*=\eta$ if $0\le \eta <\eta ^*=V_1-\frac{h_{1}^*}{h_{2}^*}V_2\approx 4118$ and $X^*=V_1-h_1^*(V_1+V_2-\eta )$ if $\eta \ge \eta ^*$. Indeed, the latter is given by solving the linear function that passes through $(\eta ^*,\eta ^*)$ and $(V_1+V_2,V_1)$. These results exactly matches Theorem 4.15.

In reality, the spatial heterogeneity may not be negligible. For example, using the same parameter set as in Fig. 6a except that $a_2=0.25, 0.3$ and 0.4, we plot the curves of $X^*$ with respect to $\eta$ in Fig. 6b. As $a_2=0.25$, the difference in biting rate is small, the optimal control strategy is similar to that in the homogeneous environment except a smaller threshold value like $\eta ^*$. As $a_2$ increases to 0.3, the biting rate of patch 2 is much higher, the optimal control strategy is to cull vectors in patch 2 when $\eta$ is small and both patches when $\eta$ becomes large. As $a_2=0.4$, the heterogeneity in biting rate is large, the optimal control strategy is to cull vectors in patch 2 until they are all culled. In all these three cases, patch 1 remains the high-risk patch before control, i.e., $\mathcal {R}_0^{(1)}>\mathcal {R}_0^{(2)}$.

## Discussion

In this paper, we proposed a multi-group multi-patch vector-borne disease model to study the effects of host and vector movements on disease transmission and control. The host movement is Lagrangian (track individuals by their label), while the vector movement is Eulerian (track individuals by current location). Firstly, we derive the basic reproduction number $\mathcal {R}_0$ of the model, and analyze the global dynamic behavior of the model system. By applying the theory of monotone dynamical systems, it is shown that the disease dynamics are completely determined by $\mathcal {R}_0$, that is, if $\mathcal {R}_0 \le 1$, then the disease-free equilibrium is globally asymptotically stable, i.e., the disease goes extinct; if $\mathcal {R}_0>1$, then there is a globally asymptotically stable endemic equilibrium, i.e., the diseases eventually persists at a constant level. Secondly, we obtained lower and upper bounds of $\mathcal {R}_0$ in heterogeneous environments that do or do not depend on the residence times matrix of hosts and the migration matrix of vectors. In homogeneous environments, $\mathcal {R}_0$ is between the minimum and maximum value of the disconnected patch reproduction number $\mathcal {R}_0^{(k)}$. When a limited amount of vector control resources are available, we considered what allocation strategy can minimize the control reproduction number. Finally, based on the two-group two-patch submodel, we numerically investigated the impact of change in host residence time proportions on $\mathcal {R}_0$ and the total host infections and found that both quantities have numerous changing patterns and there is inconsistency between disease persistence and host prevalence. Inappropriate control of population mobility may reduce the persistence of disease transmission but increase the prevalence of host population. In addition, the efficacy of vector control measures is strongly affected by spatial heterogeneity.

In 1986, Dye and Hasibeder (1986) constructed a mosquito-borne disease model with only vector commuting. In a subsequent paper (Hasibeder and Dye 1988), they proved that nonhomogeneous host selection by mosquitoes results in larger or equal basic reproduction numbers compared to those obtained under uniform host selection. In other words, nonhomogeneous mixing of hosts and vectors in a homogeneous environment increases disease persistence. This phenomenon was observed numerically by Gao and Ruan (2012) based on an SEIRS-SEI type malaria model with only host migration. In 2019, Gao et al. (2019) showed that the same result holds for a multi-patch Ross–Macdonald model with host and/or vector migration, i.e., Eulerian movement. The two-patch case with only host migration was given in an earlier survey article by Gao and Ruan (2014). Recently, Gao and Cao (2024) proved the result for a multi-group multi-patch Ross–Macdonald model with host and/or vector commuting, i.e., Lagrangian movement. The current study established the result for a model with host commuting and vector migration, i.e., Lagrangian movement for hosts and Eulerian movement for vectors. The case of host migration and vector commuting has not been analyzed yet and is less biologically plausible. We believe that nonhomogeneous mixing of hosts and vectors still promotes disease persistence. Thus, all these works together constitute a unified conclusion for the spatial spread of vector-borne diseases through Lagrangian and/or Eulerian host and/or vector movement. The host population is a resource to vectors that provides blood meals. The disease persistence is minimized if the vector population follows the ideal free distribution strategy (Fretwell and Lucas 1969). As an application, to reduce the persistence of malaria or other vector-borne diseases, it is crucial to maintain a uniform distribution of vectors and hosts. Furthermore, in case of a heterogeneous environment, the basic reproduction number has lower and upper bounds that do not depend on movement if host movement is Lagrangian (Gao and Cao 2024; Hasibeder and Dye 1988), and only movement-independent lower bound if host movement is Eulerian (Gao et al. 2019).

There is much room for improvement and generalization. Like the upper bound given in Theorem 4.3, we conjecture that the lower bound of $\mathcal {R}_0$ presented in Theorem 4.2 can be improved from $\min \mathcal {R}_{ijkr}$ to $\min \mathcal {R}_{ijkj}$. There may exist a movement-independent lower bound of $\mathcal {R}_0$ in terms of $\tilde{\mathcal {R}}_{ijk}$. In homogeneous environments, it is interesting to find the necessary and sufficient conditions for $\mathcal {R}_0(m/n)=\mathcal {R}_0(1/1)$. Some sufficient conditions, i.e., $h_k^*=v_k^*$ for all $k\in \Omega _v$ (uniform distribution of hosts and vectors), or $m=1$ (one host group), or $n=1$ (one vector patch), are given in Corollary 4.4. However, Remark 4.8 suggests that the equality can hold under other conditions. This differs from the cases with Eulerian host and/or vector movements (Gao et al. 2019) or only vector movement (Hasibeder and Dye 1988). Optimizing vector control strategy with respect to the reproduction number is a biologically meaningful but mathematically challenging problem. We only solved a very special case, that is, two-group two-patch with host movement in a homogeneous environment. The dependence of the basic reproduction number and the total number of infected hosts on population movement requires rigorous mathematical analysis, which is important for understanding the relation between disease persistence and disease prevalence. Similar to diffusion rate in Eulerian movement, we may figure out a common parameter for Lagrangian movement. In the model analysis, the irreducibility of the migration matrix of vectors leads to no vector-free patch. Due to climatic factors or vector control measures, some places where hosts live or visit may have few or no vectors. When extrinsic and intrinsic incubation periods are taken into consideration, Gao and Ruan (2012) found that host migration can drive disease from persistence to extinction in a homogeneous environment, totally differing from the model without incubation periods. Under what spatial and temporal conditions the main conclusions of the present study remain true will be the focus of our future research.
